# Insights into the Immunological Properties of Intrinsically Disordered Malaria Proteins Using Proteome Scale Predictions

**DOI:** 10.1371/journal.pone.0141729

**Published:** 2015-10-29

**Authors:** Andrew J. Guy, Vashti Irani, Christopher A. MacRaild, Robin F. Anders, Raymond S. Norton, James G. Beeson, Jack S. Richards, Paul A. Ramsland

**Affiliations:** 1 Centre for Biomedical Research, Burnet Institute, Melbourne, Australia; 2 Department of Immunology, Monash University, Melbourne, Australia; 3 Department of Medicine, University of Melbourne, Melbourne, Australia; 4 Medicinal Chemistry, Monash Institute of Pharmaceutical Sciences, Monash University, Parkville, Australia; 5 Department of Biochemistry and Genetics, La Trobe Institute for Molecular Science, La Trobe University, Melbourne, Australia; 6 Department of Microbiology, Monash University, Melbourne, Australia; 7 Victorian Infectious Diseases Service, Royal Melbourne Hospital, Melbourne, Australia; 8 Department of Surgery Austin Health, University of Melbourne, Heidelberg, Australia; 9 School of Biomedical Sciences, CHIRI Biosciences, Faculty of Health Sciences, Curtin University, Perth, Australia; New York State Dept. Health, UNITED STATES

## Abstract

Malaria remains a significant global health burden. The development of an effective malaria vaccine remains as a major challenge with the potential to significantly reduce morbidity and mortality. While *Plasmodium* spp. have been shown to contain a large number of intrinsically disordered proteins (IDPs) or disordered protein regions, the relationship of protein structure to subcellular localisation and adaptive immune responses remains unclear. In this study, we employed several computational prediction algorithms to identify IDPs at the proteome level of six *Plasmodium* spp. and to investigate the potential impact of protein disorder on adaptive immunity against *P*. *falciparum* parasites. IDPs were shown to be particularly enriched within nuclear proteins, apical proteins, exported proteins and proteins localised to the parasitophorous vacuole. Furthermore, several leading vaccine candidates, and proteins with known roles in host-cell invasion, have extensive regions of disorder. Presentation of peptides by MHC molecules plays an important role in adaptive immune responses, and we show that IDP regions are predicted to contain relatively few MHC class I and II binding peptides owing to inherent differences in amino acid composition compared to structured domains. In contrast, linear B-cell epitopes were predicted to be enriched in IDPs. Tandem repeat regions and non-synonymous single nucleotide polymorphisms were found to be strongly associated with regions of disorder. In summary, immune responses against IDPs appear to have characteristics distinct from those against structured protein domains, with increased antibody recognition of linear epitopes but some constraints for MHC presentation and issues of polymorphisms. These findings have major implications for vaccine design, and understanding immunity to malaria.

## Introduction

Intrinsically disordered proteins (IDPs) are an important class of proteins characterised by a high degree of flexibility and lack of a well-defined three-dimensional structure [1]. They have been shown to play significant roles in many cellular processes, including protein-ligand binding, DNA and RNA binding, and as flexible linkers [2–5]. Other roles for IDPs relate directly to their entropic properties, such as their proposed functions as molecular springs or in the timing of molecular processes (entropic clocks) [6–9]. Whilst many studies have examined the functional roles of disordered proteins, their immunogenic and antigenic properties have received relatively little attention.

Computational studies have shown a higher proportion of IDPs in the proteomes of eukaryotic species as compared to prokaryotes [10–12], with the proteomes of apicomplexan parasites being particularly enriched in IDPs [13]. Of the apicomplexan parasites that infect human hosts, *Plasmodium falciparum* is responsible for the highest number of deaths worldwide, although other species including *P*. *vivax* also contribute significantly to the global malaria disease burden [14]. There is an urgent need for an effective malaria vaccine, and a major challenge is to identify key antigens that are targeted by protective immune responses and to design vaccine constructs that generate highly effective and long-lasting immunity. Several current vaccine candidates for *P*. *falciparum* malaria such as CSP, MSP2, MSP3, EBA-175 RIII-V and SERA5 are targets of functional antibody responses [15–20] and are composed partly or almost entirely of disordered regions [16,21–27].

IDPs contain a number of features that may affect adaptive immune responses against *Plasmodium* spp. Firstly, the reduced proportion of bulky, hydrophobic residues in IDPs [12,28] has potential implications for peptide binding to MHC class I and II molecules, as highlighted by recent work suggesting that disordered regions across a number of species contain a paucity of MHC-binding peptides [29]. Secondly, tandem repeat regions are thought to be prevalent within IDPs, with evidence suggesting that evolution of IDPs is sometimes driven by expansion of tandem repeat regions [30,31]. Tandem repeat regions have the potential to be immunodominant [32–34] (e.g. in the sequence of the RTS, S vaccine), with certain repeat motifs capable of inducing both T-cell-dependent and T-cell-independent B-cell responses [35–37]. Finally, the occurrence of non-synonymous single nucleotide polymorphisms (SNPs) in some *P*. *falciparum* genes has been linked to immune selection pressure [38–40], with evidence from other organisms suggesting that positive selection of non-synonymous SNPs occurs at a higher rate within IDPs [41].

We hypothesised that IDPs are likely to represent major immune targets in *P*. *falciparum* and are likely to be important vaccine candidates. We sought to determine if characteristics that have been observed for IDPs of other organisms were also found in IDPs of *P*. *falciparum* and to ascertain the relevance of these characteristics in vaccine construct design. Using a variety of computational techniques, we established that IDPs within the *P*. *falciparum* proteome are abundant in immunologically-exposed subcellular locations and contain a high proportion of linear B-cell epitopes. We also determined that IDPs have a reduced proportion of MHC-binding peptides compared with ordered domains, which may adversely affect T-cell help. They also have a higher proportion of tandem repeats and polymorphisms, creating additional, but not insurmountable challenges for vaccine construct design. This study has significant implications for understanding the generation of adaptive immune responses, either through natural exposure or vaccination against IDPs, and the development of bioinformatics tools to assist in the development of future vaccine constructs.

## Results

**Fig 1:**
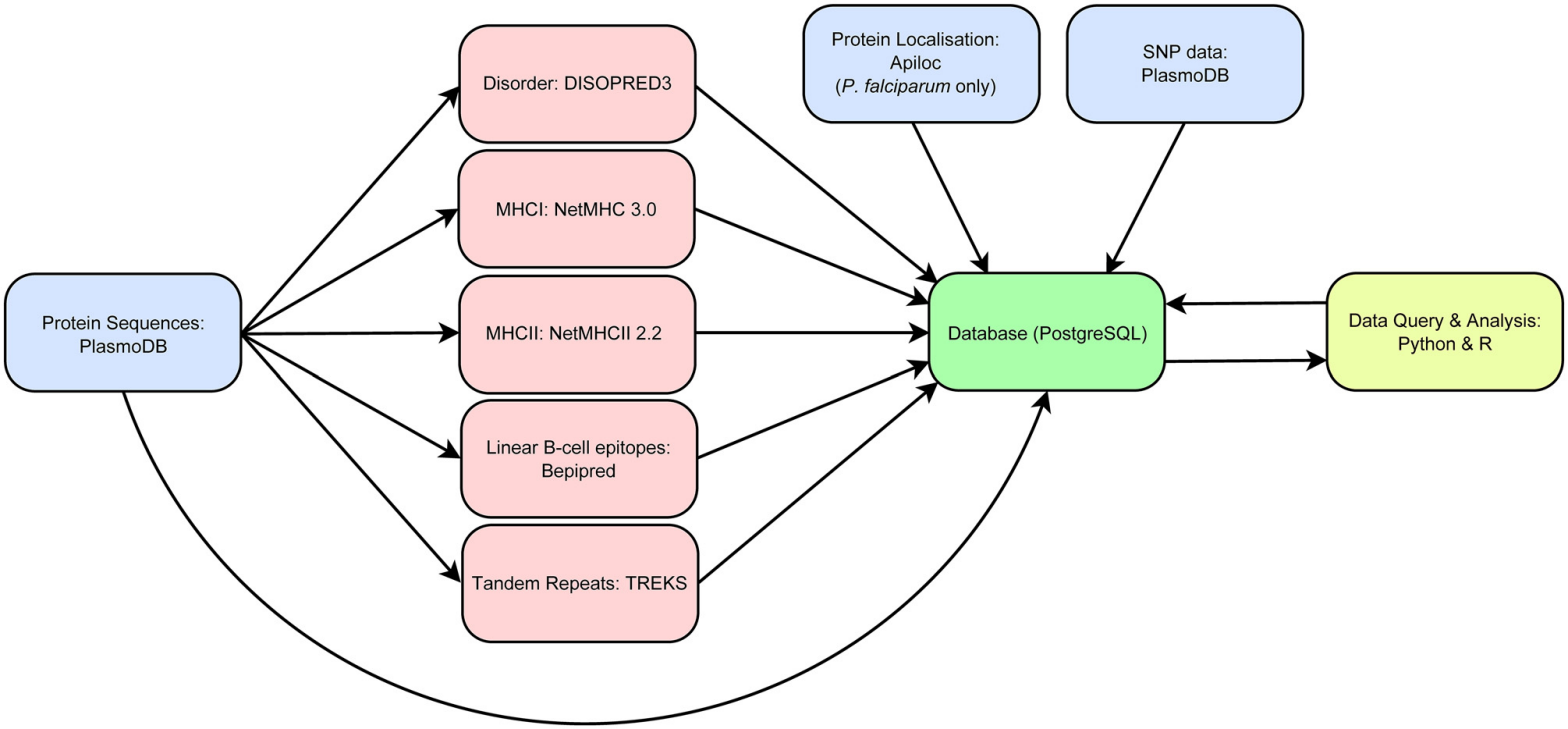
Computational workflow used for analysis of the proteome of *Plasmodium* spp. Protein coding sequences were obtained from PlasmoDB, and submitted to predictors of protein disorder, MHC binding, linear B-cell epitopes and tandem repeats. Protein localisation data for *P*. *falciparum* was obtained from ApiLoc and non-synonymous single nucleotide polymorphisms (SNPs) were obtained from PlasmoDB. All data were stored in a local PostgreSQL database and queried using custom Python scripts. Statistical analysis and data visualisation were performed using the R statistical computing package.

Sequences from the entire *P*. *falciparum* proteome were interrogated using established predictors of protein disorder, MHC class I and II binding, linear B-cell epitopes and tandem repeat regions. Information on protein localisation for *P*. *falciparum* was obtained from ApiLoc [42] and single nucleotide polymorphisms (SNPs) were obtained from PlasmoDB. Protein sequences for other *Plasmodium* spp. capable of infecting humans (*P*. *vivax* and *P*. *knowlesi*) and mice (*P*. *berghei*, *P*. *chabaudi*, and *P*. *yoelii*) were also assessed using these predictors to enable comparison across *Plasmodium* spp. Results from these predictors were stored in a local PostgreSQL database and subjected to further analysis using custom Python and R scripts (Fig 1).

### High proportions of the Plasmodium proteome are intrinsically disordered

**Fig 2:**
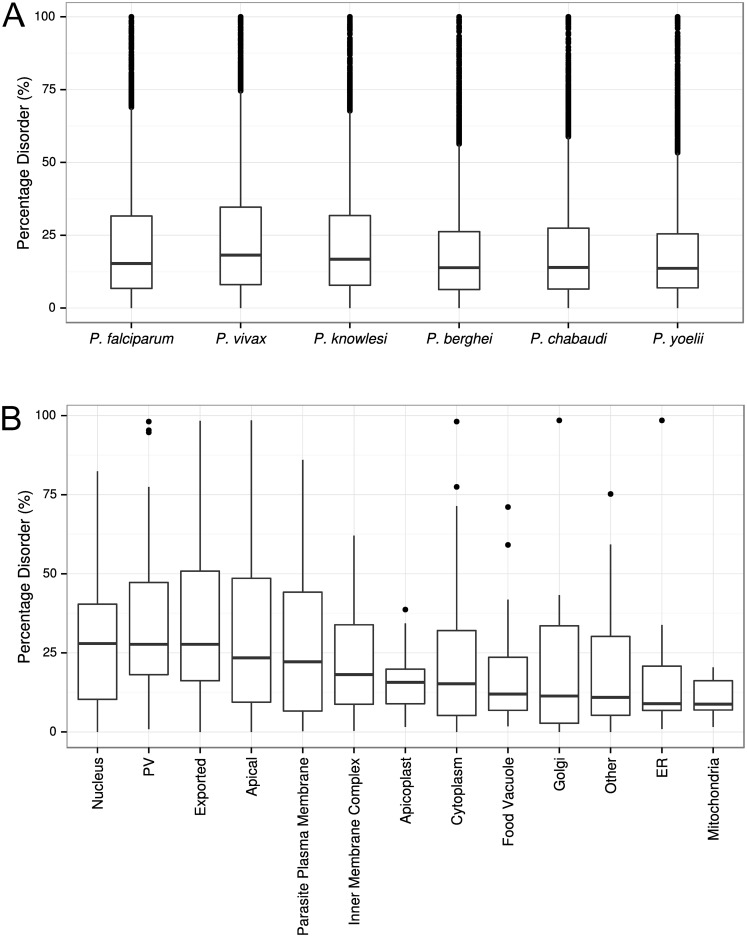
Prediction of protein disorder for various *Plasmodium* spp. and within subcellular locations for *P*. *falciparum*. **A)** Distribution of protein disorder within the proteome of each *Plasmodium* spp. at the level of individual proteins. **B)** Prediction of protein disorder for *P*. *falciparum* proteins according to subcellular localisation. Protein localisation was classified using the ApiLoc resource. A total of 451 proteins were assigned a location. Percentage disorder was calculated as the proportion of residues predicted to be disordered at the level of individual proteins. Prediction of disorder was performed using DISOPRED3.

**Fig 3:**
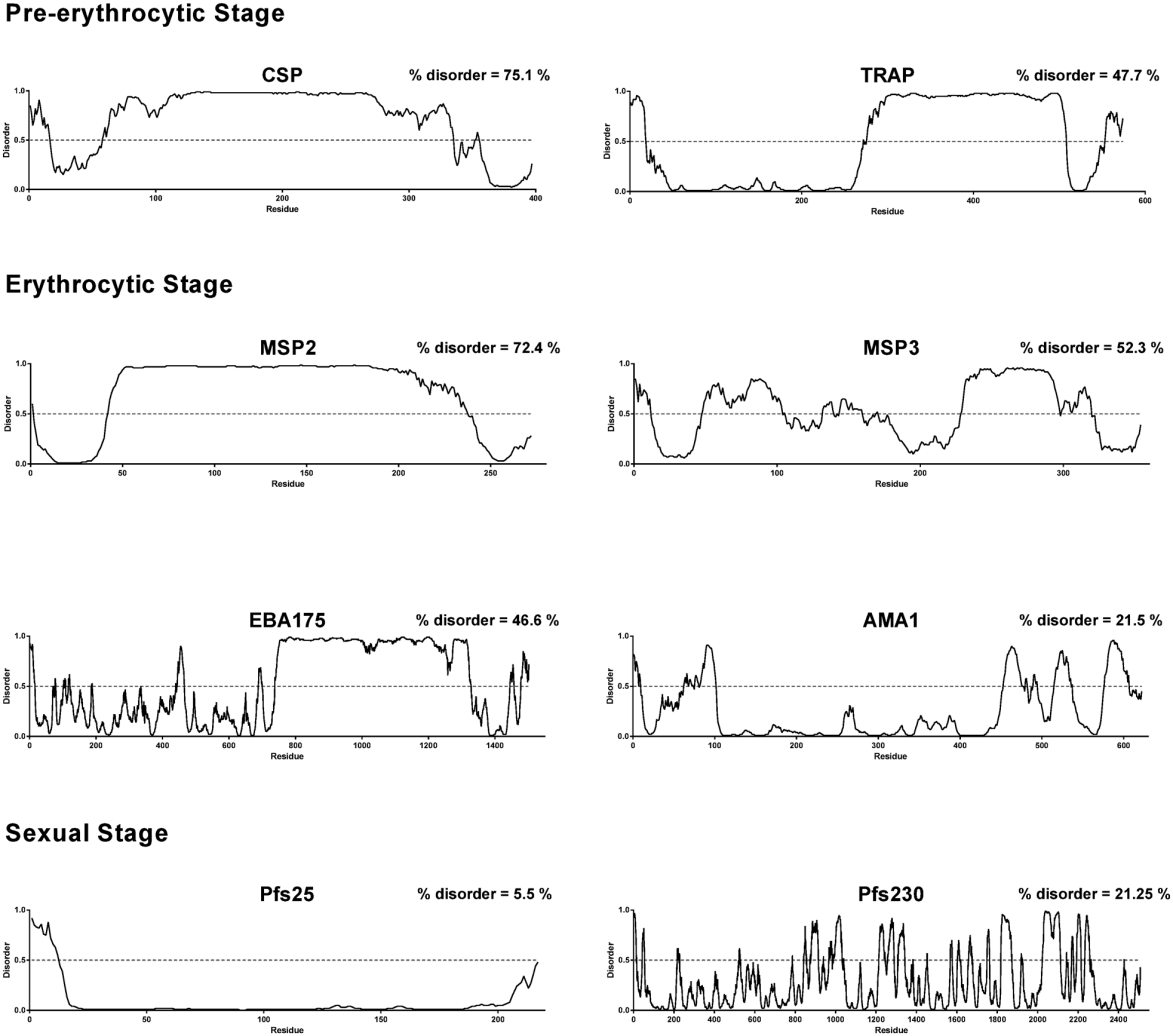
Predicted protein disorder for a number of leading *P*. *falciparum* vaccine candidates. Disorder predictions were performed using DISOPRED3. A disorder score above 0.5 is indicative of a disordered region (dashed line). All sequences used were from the *P*. *falciparum* 3D7 strain.

We considered disorder at both a per-proteome level (i.e. the number of residues across the proteome that fall within disordered regions; expressed as a proportion of residues for the entire proteome) and at a per-protein level (the percentage of predicted disordered residues for each protein). IDPs constituted a significant proportion of the proteomes of the six *Plasmodium* species assessed. On a per-proteome basis, the proportions of the proteomes predicted to be disordered were as follows: *P*. *falciparum* 32.7%, *P*. *vivax* 33.2%, *P*. *knowlesi* 30.6%, *P*. *berghei* 26.7%, *P*. *chabaudi* 27.6% and *P*. *yoelii* 27.5%. The median degree of disorder per-protein for *P*. *falciparum* was 15.5% (IQR = 6.7–31.6%; Fig 2A). No significant differences between the proportion of disorder per-protein were observed among any of the *Plasmodium* spp. tested (p > 0.05, Kruskal-Wallis rank sum test). After combining the results for the six *Plasmodium* spp. tested, the median disorder per-protein was 15.1% (IQR = 7.0–29.7%). Several leading *P*. *falciparum* vaccine candidates were also assessed to determine the proportion of these proteins that are disordered. There was a significant proportion of disorder among many of these proteins including: 1) pre-erythrocytic antigens: CSP (75.1%), LSA1 (40.6%), TRAP (47.7%); 2) erythrocytic stage antigens: MSP1 (59.1%), MSP2 (72.4%), MSP3 (52.3%), EBA175 (46.6%), AMA1 (21.5%), RESA (50.5%), Rh5 (8.0%), GLURP (95.4%), SERA5 (29.1%); and 3) sexual stage antigens: Pfs25 (5.5%) and Pfs230 (21.3%). The distribution of this disorder is shown for some selected examples, highlighting the heterogeneity of disorder amongst leading vaccine candidates, and demonstrating that vast regions of some these proteins may be almost entirely disordered (Fig 3).

### Disordered proteins are abundant within apical organelles, parasitophorous vacuole, exported proteins and the nucleus

Protein localisation data for 451 *P*. *falciparum* proteins were obtained from the curated ApiLoc database [42], but there was very limited protein localisation data for other *Plasmodium* spp. The prevalence of per-protein disorder in various subcellular locations was highest in nuclear proteins (median = 28.0%), parasitophorous vacuole (PV) proteins (median = 27.7%), exported proteins (median = 27.7%) and apical proteins (median = 23.4%). Median protein disorder was lowest in the endoplasmic reticulum proteins (median = 8.9%) and mitochondrial proteins (median = 8.8%). (Fig 2B, S1 Table). All of these values were significantly different from the median degree of disorder across the whole proteome (p<0.001 for each, Wilcoxon rank-sum test).

### Disordered proteins contain a biased amino acid composition

**Fig 4:**
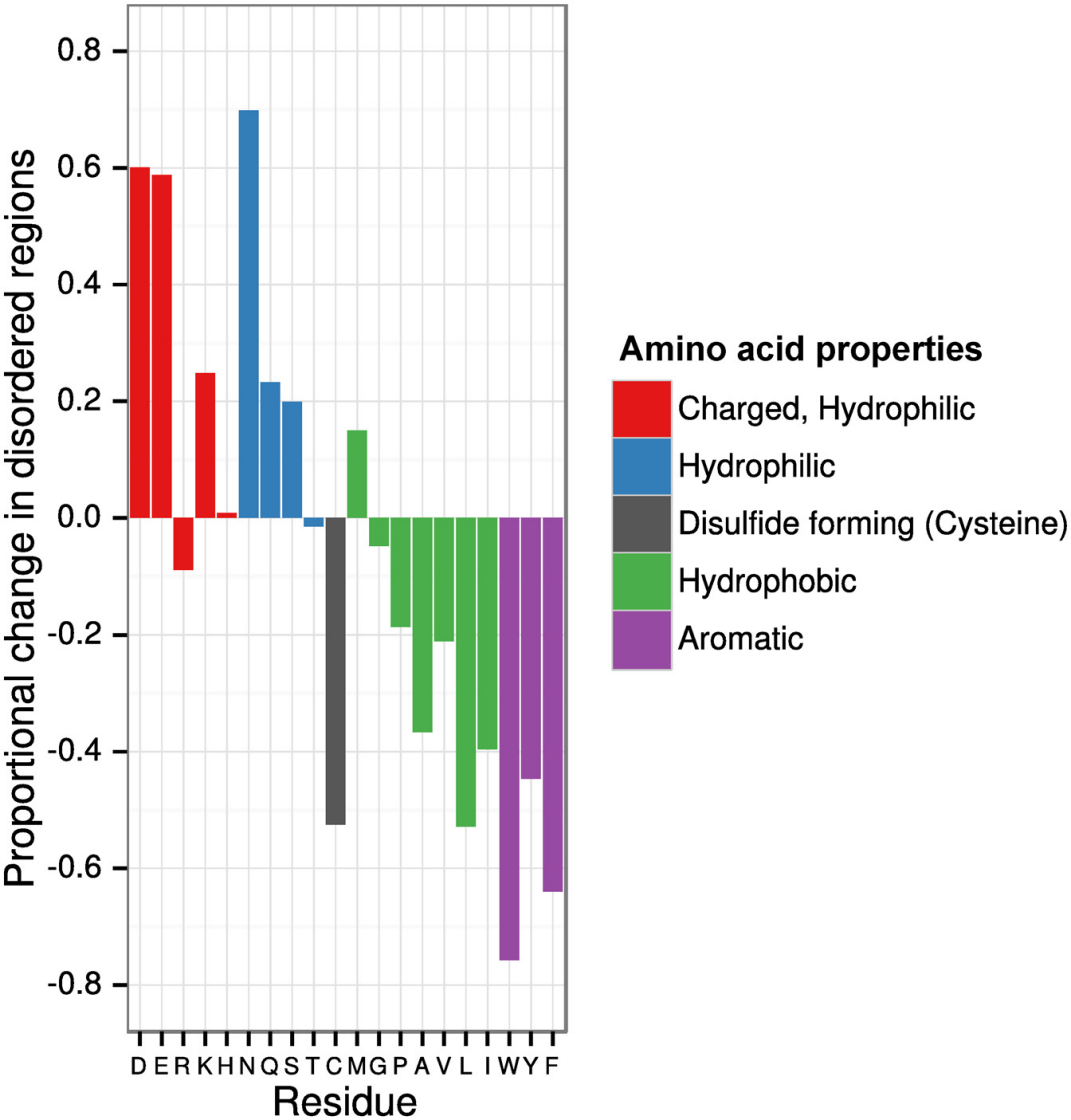
Intrinsically disordered protein domains contain a biased amino acid composition. The relative proportional change in amino acid frequency within disordered regions was calculated for the entire *P*. *falciparum* proteome, with comparison made to all predicted ordered regions within the proteome.

It has been shown previously that the amino acid composition of IDPs is distinct from that of structured proteins [12,28]. An assessment of the amino acid composition of ordered and disordered regions in the *P*. *falciparum* proteome revealed a marked reduction in aromatic residues tryptophan (W), tyrosine (Y) and phenylalanine (F), with a 76%, 45% and 64% reduction respectively. There was also a reduction in hydrophobic residues proline (P), alanine (A), valine (V), leucine (L), and isoleucine (I) in IDPs. Cysteine (C) was also significantly under-represented within disordered domains, with a 53% reduction relative to ordered regions. There was a corresponding increase within disordered regions in the proportion of charged or hydrophilic residues including aspartic acid (D), glutamic acid (E), lysine (K), asparagine (N) and glutamine (Q), with D, E and N being increased at least 50% relative to ordered regions (Fig 4).

### Disordered proteins contain fewer predicted MHC binding peptides

**Fig 5:**
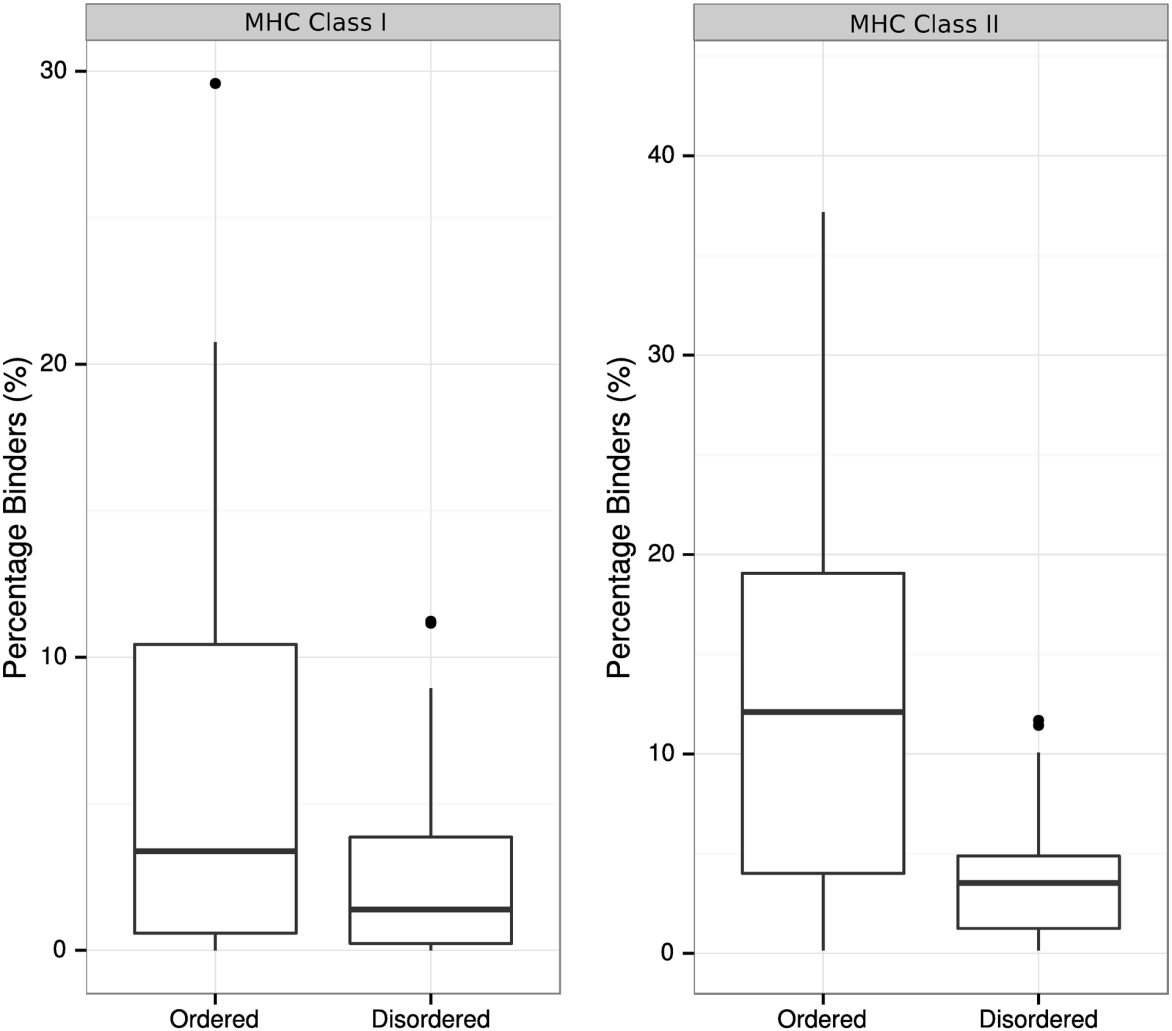
Reduced MHCI and MHCII binding in disordered proteins for *P*. *falciparum*. The proportion of peptides with predicted high affinity to MHCI and MHCII is significantly higher for peptides within an ordered protein domain. Boxplots represent the distribution of MHC-binding peptides across all MHC alleles tested.

To assess the effect of protein disorder on the predicted presentation of *P*. *falciparum* peptides via MHC class I and MHC class II, we employed *in silico* prediction of peptide binding to MHC. For each HLA allele, we defined the proteome coverage as the percentage of residues across the *P*. *falciparum* proteome that are part of a predicted high-affinity peptide (IC_50_ < 50nM). The median coverage of high-affinity peptides across all HLA alleles was then calculated. For MHC class I, the median coverage was 3.3% and 1.4% within ordered and disordered regions, respectively (p<0.0001, Wilcoxon rank sum test), equating to a ~2.3-fold decrease within disordered regions (Fig 5). For MHC class II, which is especially important for effective antibody responses, the median coverage was 12.1% and 3.5% within ordered and disordered regions, respectively (p<0.0001, Wilcoxon rank sum test), equating to a ~3.5-fold decrease within disordered regions (Fig 5). When lowering the positive threshold to include predictions for both high and low MHC affinity (predicted IC_50_ < 500nM), the median coverage for MHC class I was 17.5% within ordered regions and 6.1% within disordered regions (p<0.0001, Wilcoxon rank sum test), while the median coverage for MHC class II was 42.3% within ordered regions and 15.1% within disordered regions (p<0.0001, Wilcoxon rank sum test). When individual HLA haplotypes were assessed, decreased MHC class I and MHC class II epitopes in disordered proteins were consistently observed compared with ordered proteins for each haplotype (S1 Fig). These findings presumably reflect the reduced proportion of hydrophobic and aromatic residues within disordered domains, resulting in a reduced ability to bind MHC molecules with high affinity. There was also considerable heterogeneity observed in predicted affinities between different haplotypes, which may have implications for immunity in genetically diverse populations.

To assess the possibility of biased MHC binding within different subcellular compartments, we examined the proportion of MHC class I and MHC class II binding peptides within the subset of proteins described in the ApiLoc database (S2 Fig). Peptides were grouped according to protein disorder and subcellular protein location. No significant difference in MHC class I or MHC class II binding was observed between subcellular locations for high-binding peptides (p>0.05, Kruskal-Wallis rank sum test).

### Reduced MHC binding reflects biased amino acid composition at key peptide anchor points

**Fig 6:**
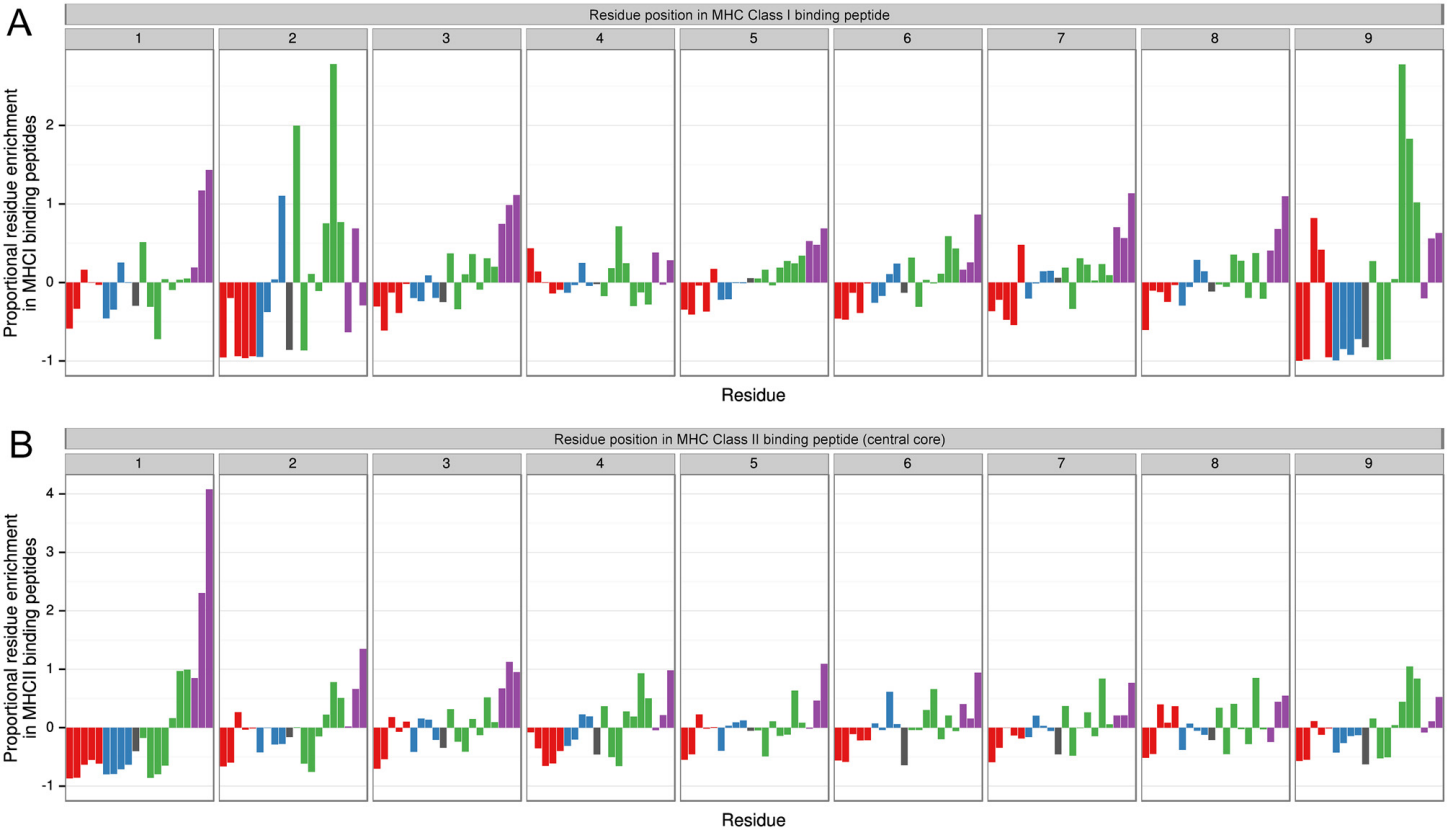
Position-specific enhancement or depletion of residues in MHC binding peptides. Residue abundance in MHC class I **(A)** and MHC class II **(B)** binding peptides was calculated relative to the abundance in the background proteome. This calculation was performed for residues at each position in an MHC binding peptide. Adjustment was made for differing sequence composition in disordered *versus* ordered regions, with results presented as a weighted average across all regions. Amino acid residue labels are omitted for visual clarity—refer to Fig 4 for residue order and colouring.

To identify potential sequence determinants that affect binding to MHC class I and MHC class II molecules, we analysed the position-dependent sequence composition of predicted high-affinity peptides (Fig 6). Peptides were classified as being part of an ordered region, a disordered region, or on the boundary of the two. Inherent differences in sequence composition between ordered and disordered regions (Fig 4) were taken into account when calculating the proportional enrichment of each residue in MHC-binding peptides (see Methods), with data presented as a weighted average across all regions (disordered/ordered/mixed). For MHC class I binding peptides, it was observed that sequence composition differed most from background levels at positions 2 and 9 of all predicted binding peptides (Fig 6A). Importantly, greater than 100% enrichment was observed for methionine (M) and leucine (L) at position 2, and arginine (R), valine (V) and leucine (L) at position 9. There was also a tendency for aromatic residues (W, Y and F) to be enriched at most other peptide positions. For MHC class II binding peptides, we considered only the predicted central core-binding region (as defined by the NetMHCII algorithm [43,44]). It was observed that sequence composition differed most from background levels at position 1 of predicted core-binding regions (Fig 6B). Phenylalanine (F) and tyrosine (Y) were particularly enriched at this position, with enrichment of leucine (L), isoleucine (I) and tryptophan (W) observed to a lesser extent. Residues that are enriched within MHC binding peptides are generally found at lower frequency within disordered regions (S3 Fig); for example, aromatic residues such as F and Y are found at much lower frequency within disordered regions, while being present at much higher frequency in position 1 of predicted MHC class II binding peptides. Similarly, hydrophilic residues are found very rarely at position 1 of MHC class II binding peptides, yet are generally enriched within disordered regions.

To determine if the position of amino acid residues within the MHC binding regions or the amino acid residue characteristics themselves biased these results, we scrambled sequences from within disordered and ordered regions, and submitted scrambled sequences to predictors of MHCI and MHCII binding. There was a small but statistically significant difference between observed MHC binding for the actual sequences, and that of the scrambled sequences (with the exception of MHCII binding within ordered regions) (S4 Fig). These shifts were small compared to the large bias in MHC binding between ordered and disordered regions, suggesting that this bias in MHC binding between regions is predominantly due to sequence composition alone, rather than the result of selective pressure or other functional/structural requirements of MHC-peptide interactions.

### Linear B-cell epitopes are more abundant in disordered proteins

**Fig 7:**
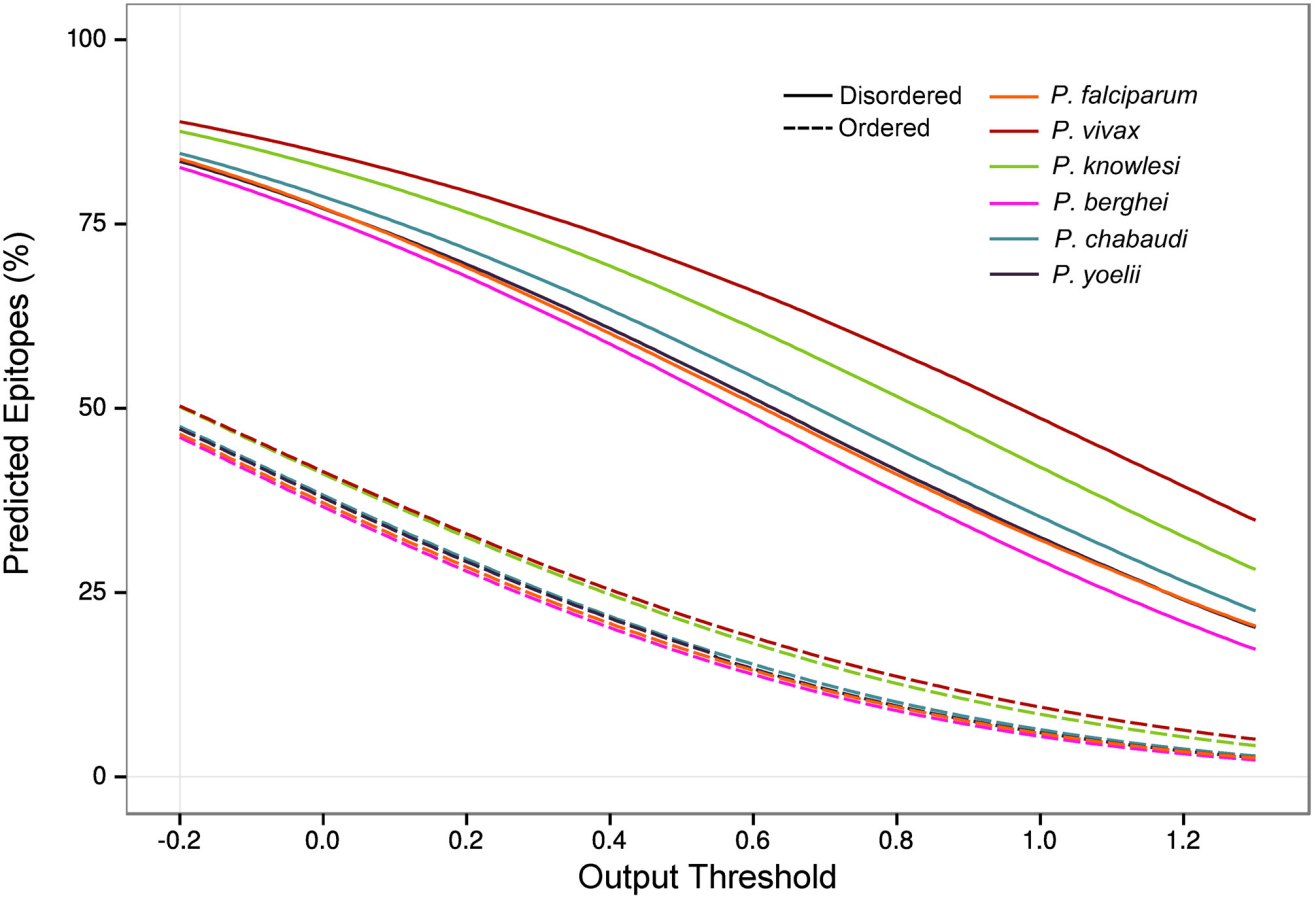
The proportion of predicted linear B-cell epitopes was higher in IDPs for all *Plasmodium* spp. Classification of disorder was achieved using DISOPRED3. BepiPred was used for prediction of linear B-cell epitopes. The number of predicted linear B-cell epitopes as a percentage of all residues is shown across a range of BepiPred output thresholds. The corresponding sensitivity/specificity for each output threshold is given at http://www.cbs.dtu.dk/services/BepiPred/output.php. Thresholds range from -0.2 (sensitivity = 0.75, specificity = 0.5) to 1.3 (sensitivity = 0.13, specificity = 0.96).

The occurrence of linear B-cell epitopes was predicted across the *P*. *falciparum* proteome and compared to the occurrence of predicted disorder, with comparison made at a per-residue level. The proportion of residues predicted to contain linear B-cell epitopes was assessed with the BepiPred algorithm using a range of different output thresholds that reflect the sensitivity and specificity of detecting a linear B-cell epitope (i.e. lower output threshold has high sensitivity but low specificity, and high output thresholds have low sensitivity but high specificity). Across all these output thresholds, linear B-cell epitopes were predicted to be more common in regions of disorder than in structurally ordered regions (Fig 7; solid line and dashed lines respectively). A comparison with other *Plasmodium* spp. showed that *P*. *vivax* contained the highest proportion of predicted linear B-cell epitopes, for both ordered and disordered regions, whereas *P*. *falciparum* contained the second lowest proportion.

When examining the distribution of predicted linear B-cell epitopes within various subcellular compartments, proteins localised to the PV, parasite plasma membrane proteins, exported proteins, apical proteins and nuclear proteins all had a significantly higher percentage of predicted linear B-cell epitopes compared to the background proteome (p < 0.001 for all except parasite plasma membrane, p = 0.005; Wilcoxon rank sum test). Residues were then grouped according to predicted protein disorder, as predicted linear B-cell epitopes were correlated with predicted disorder. Levels of predicted linear B-cell epitopes remained significantly higher in PV proteins, exported proteins and nuclear proteins after accounting for protein disorder (S5 Fig and S2 Table).

### Tandem repeat regions are more common in disordered proteins

**Fig 8:**
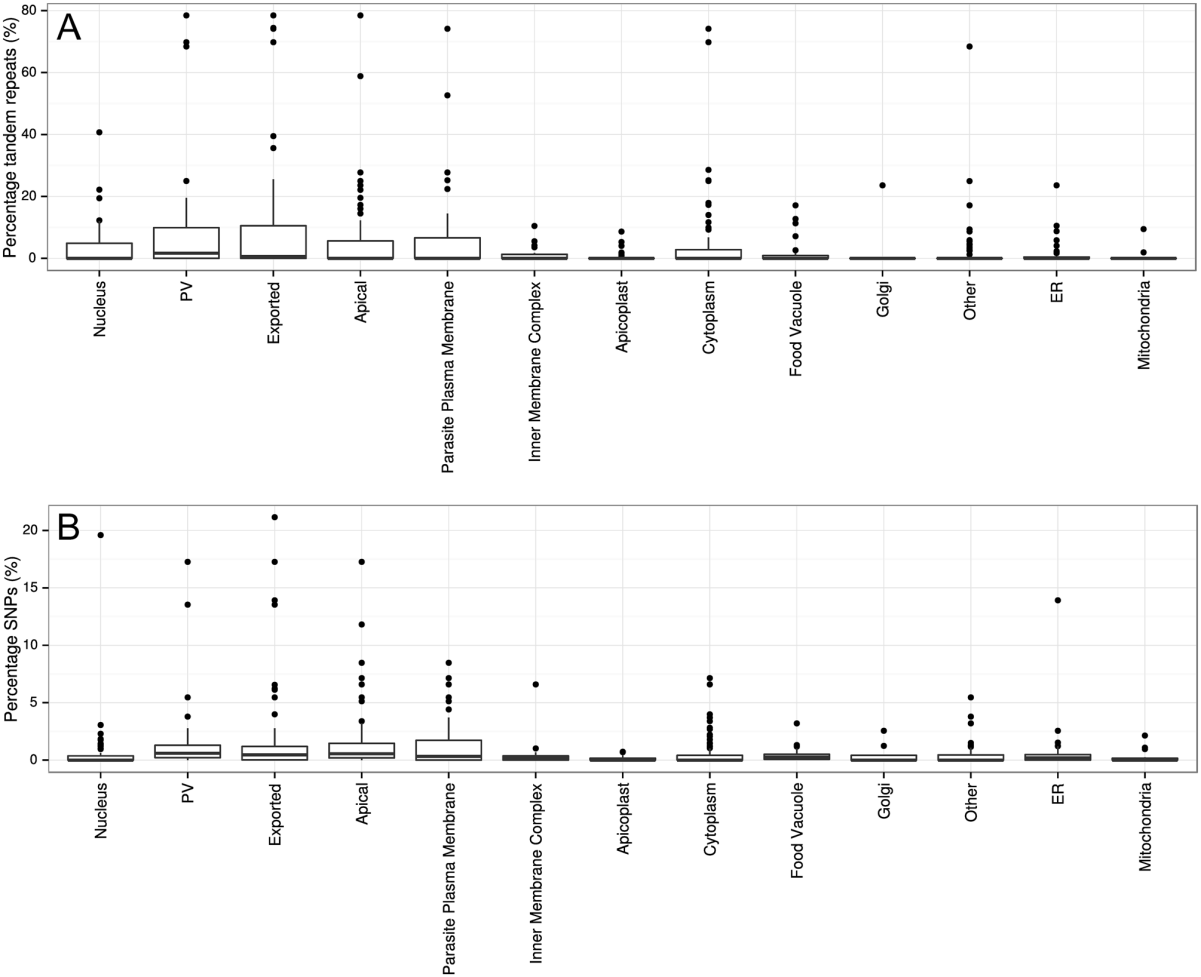
Distribution of tandem repeats and non-synonymous SNPs within different subcellular compartments for *P*. *falciparum*. **(A)** Prediction of tandem repeats was performed using T-REKS. Percentage tandem repeats was defined as the percentage of each protein that contains tandem repeat sequences. **(B)** Percentage SNPs was calculated as the percentage of residues within each protein that contain identified SNPs with a minimum minor allele frequency of 5%. Protein localisation was classified using the ApiLoc resource. A total of 451 proteins were assigned a location.

The occurrence of tandem repeat sequences within the *P*. *falciparum* proteome and the relationship to regions of structural disorder was examined to assess the potential role of IDPs in the generation of immunodominant antibody responses. Tandem repeat sequences were identified using T-REKS [45] with a Psim cut-off of 0.8. When grouped according to protein disorder, tandem repeats make up 1.7% of ordered regions, compared to 12.9% of disordered regions. Of all the identified tandem repeat regions, 79% fell within predicted disordered regions. To assess potential bias in the occurrence of tandem-repeat domains within different subcellular compartments, we analysed the occurrence of tandem repeats for the subset of proteins in the ApiLoc database (Fig 8A). Compared to the total *P*. *falciparum* proteome, exported proteins (p = 0.004) and proteins localised to the PV (p = 0.02) had a significantly higher percentage of tandem repeats (Wilcoxon rank-sum test). Lower levels of tandem repeats were observed in proteins in the cytoplasm (p = 0.01), endoplasmic reticulum (p = 0.03), apicoplast (p = 0.001) and mitochondria (p < 0.001) (S3 Table).

### Non-synonymous single nucleotide polymorphisms are more common in disordered proteins

The occurrence of non-synonymous SNPs in the *P*. *falciparum* proteome was analysed, with residues grouped according to predicted protein disorder. The percentage of disordered regions that are polymorphic was 2.5%, compared to 1.0% of ordered regions (p < 0.001, Pearson’s chi-squared test). When proteins were grouped according to subcellular location, an increased proportion of SNPs (as compared to the *P*. *falciparum* proteome) was observed in exported proteins (p = 0.001, Wilcoxon rank sum test), proteins localised to the PV (p = 0.001, Wilcoxon rank sum test) and apical proteins (p < 0.0001, Wilcoxon rank sum test) (Fig 8B, S4 Table).

## Discussion

The last few decades have seen an increased understanding of the role of IDPs in various biological systems and an appreciation of their functional importance. Numerous experimental techniques have been employed to identify and characterise IDPs [46], and these have been complemented by a number of computational algorithms developed to predict the occurrence of protein disorder using protein sequence data [47]. Little work has been performed examining the immunological properties of this class of protein, perhaps due to the relative scarcity of IDPs in most bacteria and viruses [10,11]. Eukaryotic organisms, however, are known to contain a relative abundance of IDPs, with apicomplexan parasites including *Plasmodium* and *Toxoplasma* spp. being particularly enriched in IDPs [13]. It is therefore important to understand the potential differences in immune recognition of IDPs compared to ordered protein domains. In this study, we applied a number of computational prediction algorithms at a proteomic level to gain further insight into the role of IDPs as potential antigenic targets.

A high proportion of the *P*. *falciparum* proteome was predicted to be disordered and this was also the case for other *Plasmodium* spp. IDPs appear to be especially enhanced in apical and exported proteins, suggesting that they may play functional roles in parasite invasion and sequestration, and that they are also likely to be accessible to antibody recognition on intact parasites. Almost nothing is known about the actual functional role of IDPs in *Plasmodium* spp. It is possible that they may play a role as flexible linkers between ordered domains, enabling rapid molecular recognition of host ligands during parasite invasion. This is potentially the case for the erythrocyte binding-like (EBL) family of proteins, which contain a disordered domain termed region III-V (RIII-V) [48]. The EBL family of proteins are found in *P*. *falciparum*, *P*. *vivax* and *P*. *knowlesi*, and includes proteins such as the Erythrocyte Binding Antigens (e.g. EBA-140, EBA-175, EBA-181) and Duffy Binding Protein (DBP). Molecular recognition of erythrocyte receptors by the EBL family of proteins occurs via an N-terminal structured domain termed region II (RII) [39,49,50]. We hypothesise that recognition and binding of erythrocyte receptors via RII is expedited by the flexibility of the adjacent RIII-V domain. Of note, antibodies to the disordered RIII-V of EBA175 can inhibit erythrocyte invasion [18,51]. Similarly, antibodies to the repeat region of CSP can inhibit sporozoite infection of hepatocytes [52], while antibodies against MSP2 can fix complement components to inhibit erythrocyte invasion [53]. These findings indicate the importance of IDPs in host cell invasion and as immune targets.

We observed a general enrichment of charged and hydrophilic residues within disordered regions of *P*. *falciparum*, with a corresponding decrease in the proportion of aromatic and hydrophobic residues. These observations are consistent with previous studies of IDPs [12,28], although enrichment of D and N was not observed in the study by Dunker *et al*. [12], while enrichment in N was not observed by Radivojac *et al*. [28]. It is important to note that neither of these studies accounted for potential biases in amino acid usage between species, which may explain the observed differences between studies. Our observed reduction in aromatic and hydrophobic residues in IDPs was noted to affect key peptide anchor points for MHC class I and class II binding, supporting recent findings by Mitic *et al*. [29]. Both MHC class I and II presentation require peptides to be anchored in the MHC binding groove through interactions with hydrophobic binding pockets and additional interactions with the floor and walls of the binding channel [54–57]. Reduced MHC class II binding is likely to reduce antigen presentation to helper CD4^+^ T-cells and the acquisition of effective antibody responses, while reduced MHC class I binding is likely to reduce antigen presentation to CD8^+^ T-cells which are required for immune responses against the liver stage infection [58,59]. MHC class I and class II epitopes were identified within IDPs, however, indicating that these potential limitations may be overcome by careful design of vaccine constructs and a detailed knowledge of the HLA haplotypes of target populations.

Using a sequence-based linear B-cell epitope prediction method [60], it was noted that predicted linear B-cell epitopes were significantly enriched within IDPs. This is not surprising considering that most of the polypeptide chain within an IDP is accessible to antibody binding. Several studies have characterised linear epitopes within IDPs [24,61–64], although there is a notable report describing the existence of a discontinuous epitope with an IDP [65]. We do note that current sequence-based prediction algorithms for linear B-cell epitopes should be used with caution as there is some concern that they perform relatively poorly compared to similar predictors for MHC binding [66]. While this could be due to inherent structural differences between antibody-antigen and MHC-peptide complexes, it has been suggested that current training datasets and classification methods for B-cell epitope predictors are inadequate [67]. Indeed, our recent study of *P*. *falciparum* MSP2 found that B-cell epitope predictors were poor predictors of individual immunogenic epitopes within this largely disordered protein [68].

It has been previously postulated that IDPs, and tandem repeat regions in particular, may play an important role in the immune evasion of various parasites including *Plasmodium* [37,69,70], *Trypanosoma* [32], *Leishmania* [33,71] and *Ehrlichia* [34]. Tandem repeat regions may induce immunodominant responses that act as immunological decoys, masking responses against functionally important epitopes. This hypothesis is consistent with our finding that a high proportion of IDPs are located in immunologically-exposed subcellular compartments and the observation that tandem repeat regions are predominantly located within IDPs. Repeat protein sequences bear some similarity to the repeated structural motifs found on bacterial polysaccharides that are known to elicit T-cell-independent type 2 B-cell responses. Although such responses have been described against polysaccharide antigens, there is evidence suggesting that some protozoan proteins contain tandem repeats that can act as T-cell-independent type 2 antigens [35–37], negating the need for CD4^+^ T-cell help. Perhaps more importantly, immunodominant responses against protein tandem repeats may develop due to the increased avidity of an antibody to a region in which identical epitopes are located at several adjacent regions of the polypeptide, increasing the apparent epitope concentration within the vicinity of bound antibody. This is likely to lead to rapid antibody re-binding upon dissociation, resulting in high antibody avidity, despite a relatively lower affinity for a single epitope site. In this way, selection of B-cells from the naïve B-cell repertoire is likely to be biased towards cells with reactivity to such tandem repeat domains.

Within *P*. *falciparum*, there is mixed evidence for the immunodominant nature of tandem repeats. For example, strong antibody responses are acquired naturally against the immunodominant NANP repeat region of CSP [72–74] that is predicted to adopt a coiled-coil structure [75] and antibodies against SERA-5 predominantly target a disordered N-terminal octamer repeat [19]. In contrast, some disordered tandem-repeats within MSP2 (3D7 allele) are poorly immunogenic, which has been attributed to a high degree of conformational flexibility compared to the rest of the sequence [68]. Taken together, protein tandem repeats appear to be immunodominant in some cases, but may be poorly immunogenic in others, possibly due to high flexibility and a large loss of conformational entropy upon antibody binding.

Our observation that IDPs contain a higher proportion of amino acid polymorphisms as a result of non-synonymous SNPs is consistent with previous studies and has important implications for vaccine design. Recent work in *Saccharomyces cerevisiae* showed that IDPs contained a higher proportion of non-synonymous SNPs, with disordered regions shown to be under weaker negative selection than ordered domains [76]. This was attributed to reduced structural constraints for disordered regions, being more tolerant to amino acid changes, especially changes to amino acids with similar characteristics. Many non-synonymous SNPs within *P*. *falciparum* appear to be maintained as a result of immune selection pressure, with many of the resulting polymorphisms located on the protein surface and hence accessible to antibody binding [77]. IDPs have a higher proportion of residues accessible for antibody binding which may contribute to the observed increase in non-synonymous SNPs to some degree. Interestingly, only a small number of genes (~100) within the *P*. *falciparum* proteome have been observed to be under balancing selection [78], and hence it is unlikely that immune pressure alone is responsible for the observed increase in polymorphic residues within IDPs [79].

### Conclusions

The role of IDPs as antigenic targets is poorly understood, despite their relative abundance in major human pathogens such as *P*. *falciparum*. We have shown here that the biased amino acid composition of IDPs can limit their presentation via MHC molecules and may influence the generation of antibody responses and B-cell memory. Furthermore, we have demonstrated that immunologically-exposed subcellular compartments within *P*. *falciparum* have a higher proportion of IDPs, a greater number of tandem repeat regions, and a higher incidence of non-synonymous SNPs. This indicates that IDPs can be involved in generating immunodominant antibody responses, and that some may play a role in immune evasion. Despite these apparent limitations, it is clear that some IDPs are targeted by functional immune responses and that some of these antigens are realistic vaccine candidates. Indeed, we have shown that a number of leading candidates contain a significant proportion of disordered regions. These findings have major implications for vaccine design, and understanding immunity to malaria.

## Methods

### Protein sequences

Protein sequences for *P*. *falciparum* (3D7), *P*. *vivax* (Sal-1) [80], *P*. *chabaudi* (chabaudi) [81], *P*. *berghei* (ANKA) [81] and *P*. *knowlesi* (Strain H) [82] were obtained from PlasmoDB [83] (http://plasmodb.org). All protein-coding sequences were selected for each organism with pseudo-genes excluded, and protein sequences downloaded in FASTA format.

### Disorder prediction

DISOPRED3 software was used for prediction of protein disorder [84], and was chosen due to its high ranking in independent benchmarking [85]. The DISOPRED3 algorithm utilises a combination of a support vector machine (SVM), artificial neural network (ANN) and nearest-neighbour classifier to classify residues as disordered/ordered. An initial PSI-BLAST search is also used to create a sequence profile that is then passed to the SVM. Generation of sequence profiles using PSI-BLAST was performed using the UniRef90 protein database and blast-2.2.26 software package from NCBI. PSI-BLAST was run with 3 passes, with an e-value threshold of 0.001 for inclusion in the multi-pass model. PSI-BLAST checkpoint file was saved and used as an input to the DISOPRED2 SVM algorithm (part of the DISOPRED3 prediction workflow). The default threshold was used for DISOPRED3, with a disorder score above 0.5 indicating predicted disorder. DISOPRED3 software is freely available and was obtained from http://bioinfadmin.cs.ucl.ac.uk/downloads/DISOPRED/ (last accessed 25/06/2015). For analysis of protein disorder, we considered disorder at both a per-proteome level (i.e. the number of residues across the proteome that fall within disordered regions; with no adjustment for protein length) and at a per-protein level (the percentage of residues within each protein predicted to be disordered). A similar approach was taken with all other predictors.

### MHC binding prediction

NetMHC 3.0 [86,87] and NetMHCII 2.2 [43,44] were used for prediction of MHC class I and II binding peptides, respectively. Peptide lengths of 9 (NetMHC) and 15 (NetMHCII) residues were used for all predictions. Peptides were grouped according to their predicted binding affinity (IC_50_): High-affinity, IC_50_ < 50nM; Low-affinity, 50 nM < IC_50_ < 500 nM; No-binding, IC_50_ >500 nM. Predictions were performed for all human HLA alleles available in each predictor. Both prediction algorithms were downloaded from http://www.cbs.dtu.dk/services/NetMHCII/ and http://www.cbs.dtu.dk/services/NetMHC/ (last accessed on 25/06/2015).

For prediction of MHC binding within scrambled sequences from *P*. *falciparum*, we obtained the predicted disorder scores for each protein, and scrambled sequences within ordered and disordered regions separately, retaining the overall disorder profile for each protein. These scrambled sequences were then submitted to predictors of MHC binding as above. This procedure was repeated four times with the scrambled proteome of *P*. *falciparum*, with results averaged between repeats.

### B-cell epitope prediction

BepiPred 1.0 was used for prediction of linear B-cell epitopes [60]. An output threshold of 0.9 was used (sensitivity = 0.25, specificity = 0.91) for identification of B-cell epitopes (unless stated otherwise). This threshold was chosen to provide a high level of certainty for predicted B-cell epitopes. For comparison of linear B-cell epitopes across *Plasmodium* spp., and between ordered and disordered regions, we used a range of output thresholds, from -0.2 (sensitivity = 0.75, specificity = 0.5) to 1.3 (sensitivity = 0.13, specificity = 0.96). Any residue with an output score above the threshold was considered to be part of a linear B-cell epitope. BepiPred software was obtained from http://www.cbs.dtu.dk/services/BepiPred/ (last accessed on 25/06/2015).

### Protein localisation

Protein localisation data were obtained from the ApiLoc database (http://apiloc.biochem.unimelb.edu.au/apiloc/apiloc) for *P*. *falciparum* sequences only (451 proteins) (last accessed on 25/06/2015). While ApiLoc also contains curated localisations for other *Plasmodium* spp., the number of proteins available for other species was too low to enable proper comparison among subcellular localisations (*P*. *berghei*, 61 proteins; *P*. *vivax*, 18 proteins; *P*. *knowlesi*, 6 proteins; *P*. *chabaudi*, 4 proteins).

### Identification of tandem repeat sequences

Tandem repeat sequences were identified using T-REKS, a program for the detection of repeat sequences based on a K-means algorithm [45]. T-REKS software was obtained from http://bioinfo.montp.cnrs.fr/?r=t-reks/ (last accessed 25/06/2015), and was run as a standalone Java program. The percentage similarity (P_sim_) threshold was set to 0.8 for all predictions, with filtering of overlapping repeats disabled. For residues that were part of overlapping repeats, only the repeat with the highest Psim value was considered.

### Analysis of amino acid substitutions due to non-synonymous point mutations

Data for single nucleotide polymorphisms from *P*. *falciparum* were downloaded from PlasmoDB. Within PlasmoDB, SNPs were identified based on differences within a group of isolates, with 3D7 chosen as the reference strain. SNPs were selected from isolates obtained from all available geographic locations. An 80% read frequency threshold was used, with a minimum minor allele frequency of 5%. To examine amino acid substitutions, only non-synonymous SNPs within coding regions of DNA were used.

### Workflow and database integration

Protein sequences in FASTA format were submitted to predictors of protein disorder, MHC binding, B-cell epitopes, and tandem repeats. Output files were collated and reduced to a format amenable to storage in an SQL database. Results were stored in a local SQL database (PostgreSQL 9.3.6). Protein localisation data from ApiLoc and SNP data were also stored in the SQL database. Sequence input to various prediction algorithms, output data processing and SQL queries were handled using in-house custom Perl and Python scripts (S1 File). The computational workflow is depicted in Fig 1.

### Sequence analysis of MHC binding peptides

As disordered and ordered regions tend to have a different amino acid composition, we accounted for the background amino acid frequency of ordered and disordered regions when examining the enrichment of particular residues within MHC-binding peptides. Peptides that fell on the boundary of disordered and ordered regions were considered to be part of a mixed region. To do this, we defined the proportional enrichment *f’*_*ijk*_ (Eq 1) for any particular residue *i* found at position *j* within all MHC binding peptides in a region *k* (disordered, ordered or mixed), such that residues that are neither enriched nor depleted were assigned a value of 0, while residues with a 100% increase/decrease in frequency were assigned a value of +/-1:

$${f^{\prime }}_{ijk}=\;\frac{n_{ijk}/\Sigma_{i}n_{ijk}}{N_{ijk}/\Sigma_{i}N_{ijk}}-1$$ (1)

where:

*n*_*ijk*_ = number of times residue *i* is found at position *j* within a predicted MHC binding peptide within region *k*.

*N*_*ijk*_ number of times residue *i* is found at position *j* within any peptide within region *k*.

Σ_*i*_ *n*_*ijk*_ indicates a sum over all residues *i* at position *j* for residues found within an MHC binding peptide within region *k*.

Σ_*i*_ *N*_*ijk*_ indicates a sum over all residues *i* at position *j* for residues found within region *k*.

To determine the average enrichment across the entire proteome for residues in MHC-binding peptides, we defined an average enrichment value *f*_*ij*_ (Eq 2) with correction for sequence composition in each region *k* as:

$$f_{ij}=(\sum_{k}\frac{n_{ijk}}{N_{ijk}/\sum_{i}N_{ijk}})/(\sum_{k}\sum_{i}n_{ijk})-1$$ (2)

This represents a weighted average of MHC binding residue enrichment across all regions *k*, with adjustment for amino acid frequency within each region *k*.

### Statistical analysis

All plots and statistical analysis were produced with the R statistical computing package (R Project for Statistical Computing, http://www.r-project.org/), with RStudio IDE v. 0.97.551 used for code development. R package ggplot2 was utilised for most plots [88].

## Supporting Information

S1 Fig Predicted MHCI and MHCII binding for the P. falciparum proteome, grouped by protein disorder. The proportion of peptides with predicted binding to MHCI (A) and MHCII (B) is significantly higher for peptides that are contained within a structured protein domain. Prediction of protein disorder was performed using DISOPRED3, while predictions of MHC class I and MHC class II binding were performed with NetMHC 3.0 and NetMHCII 2.2 Peptides were grouped according to their predicted binding affinity (IC50): High-affinity, IC50<50nM; Low-affinity, 50nM<IC50<500nM; No-binding, IC50>500nM.
(PDF)

S2 Fig Distribution of high-affinity MHC-binding peptides from *P*. *falciparum* across a range of HLA alleles, grouped according to predicted protein disorder and known protein localisation. No significant difference in the proportion of MHCI **(A)** or MHCII **(B)** binding peptides was observed between different subcellular locations (p > 0.05, kruskal-wallis rank sum test). Boxplots represent the distribution of MHC-binding peptides across all MHC alleles tested. Prediction of protein disorder was performed using DISOPRED3, while prediction of MHC class I and MHC class II binding was performed with NetMHC 3.0 and NetMHCII 2.2. Peptides with predicted high binding affinity are shown (IC50<50nM).
(PDF)

S3 Fig Residues that are enriched within MHC binding peptides are generally found at lower frequency within disordered regions. The position specific enhancement of each residue in both MHC class I **(A)** and MHC class II **(B)** binding peptides (IC50 < 50nM) was plotted against the proportional enrichment of that residue in disordered regions. Prediction of protein disorder was performed using DISOPRED3, while prediction of MHC class I and MHC class II binding was performed with NetMHC 3.0 and NetMHCII 2.2.
(PDF)

S4 Fig Predicted MHC binding for scrambled sequences from *P*. *falciparum*, compared to predicted MHC binding of native sequence. Sequences within disordered and ordered regions of each *P*. *falciparum* protein were scrambled, and the resultant scrambled proteome was submitted to predictors of MHC class I **(A)** and MHC class II **(B)** binding. Sequence scrambling was performed 4x, with results from MHC predictors averaged across all repeats. Prediction of disorder was performed with DISOPRED3.
(PDF)

S5 Fig Distribution of linear B-cell epitopes within *P*. *falciparum* proteins, grouped according to subcellular localisation and predicted protein disorder. Classification of disorder was achieved using DISOPRED3. BepiPred was used for prediction of linear B-cell epitopes. A threshold of 0.9 was used for BepiPred predictions. Protein localisation was classified using the ApiLoc resource. A total of 451 proteins were assigned a location.
(PDF)

S1 File Computational scripts used to generate data, perform analysis and generate figures. (ZIP)

S1 Table Summary statistics for predicted protein disorder of *P*. *falciparum* proteins, grouped according to subcellular localisation. Protein localisation was classified using the ApiLoc resource. Prediction of disorder was performed using DISOPRED3. A total of 451 proteins were assigned a location. Percentage disorder was calculated as the proportion of residues predicted to be disordered at the level of individual proteins.
(DOCX)

S2 Table Summary statistics for percentage linear B-cell epitopes within *P*. *falciparum* proteins, grouped according to subcellular localisation. Protein localisation was classified using the ApiLoc resource. A total of 451 proteins were assigned a location. A Wilcoxon Rank-Sum test was performed on proteins from each subcellular location, comparing the percentage of residues predicted to be part of a linear B-cell epitope for each protein in that location, to the distribution within the entire *P*. *falciparum* proteome. Residues were grouped according to predicted protein disorder, and statistical analysis applied to each group (ordered/disordered).
(DOCX)

S3 Table Summary statistics for predicted tandem repeats within *P*. *falciparum* proteins, grouped according to subcellular localisation. Protein localisation was classified using the ApiLoc resource. Prediction of tandem repeats was performed using TREKS, with a PSIM cutoff of 0.8. A total of 451 proteins were assigned a location. Percentage tandem repeats was calculated as the proportion of residues predicted to be part of a tandem repeat at the level of individual proteins. A Wilcoxon Rank-Sum test was performed on proteins from each subcellular location, comparing the percentage tandem repeats for proteins within each respective location to the distribution of percentage tandem repeats within the entire *P*. *falciparum* proteome.
(DOCX)

S4 Table Summary statistics for SNPs within *P*. *falciparum* proteins, grouped according to subcellular localisation. Protein localisation was classified using the ApiLoc resource. A total of 451 proteins were assigned a location. A Wilcoxon Rank-Sum test was performed on proteins from each subcellular location, comparing the percentage of residues targeted by non-synonymous SNPs for each protein in that location, to the distribution of SNPs within the entire *P*. *falciparum* proteome.
(DOCX)
