# Supplementary figures and images for: Insights into the Immunological Properties of Intrinsically Disordered Malaria Proteins Using Proteome Scale Predictions
Source: PLoS One. 2015 Oct 29;10(10):e0141729. doi: 10.1371/journal.pone.0141729 (PMC4626106; doi:10.1371/journal.pone.0141729)

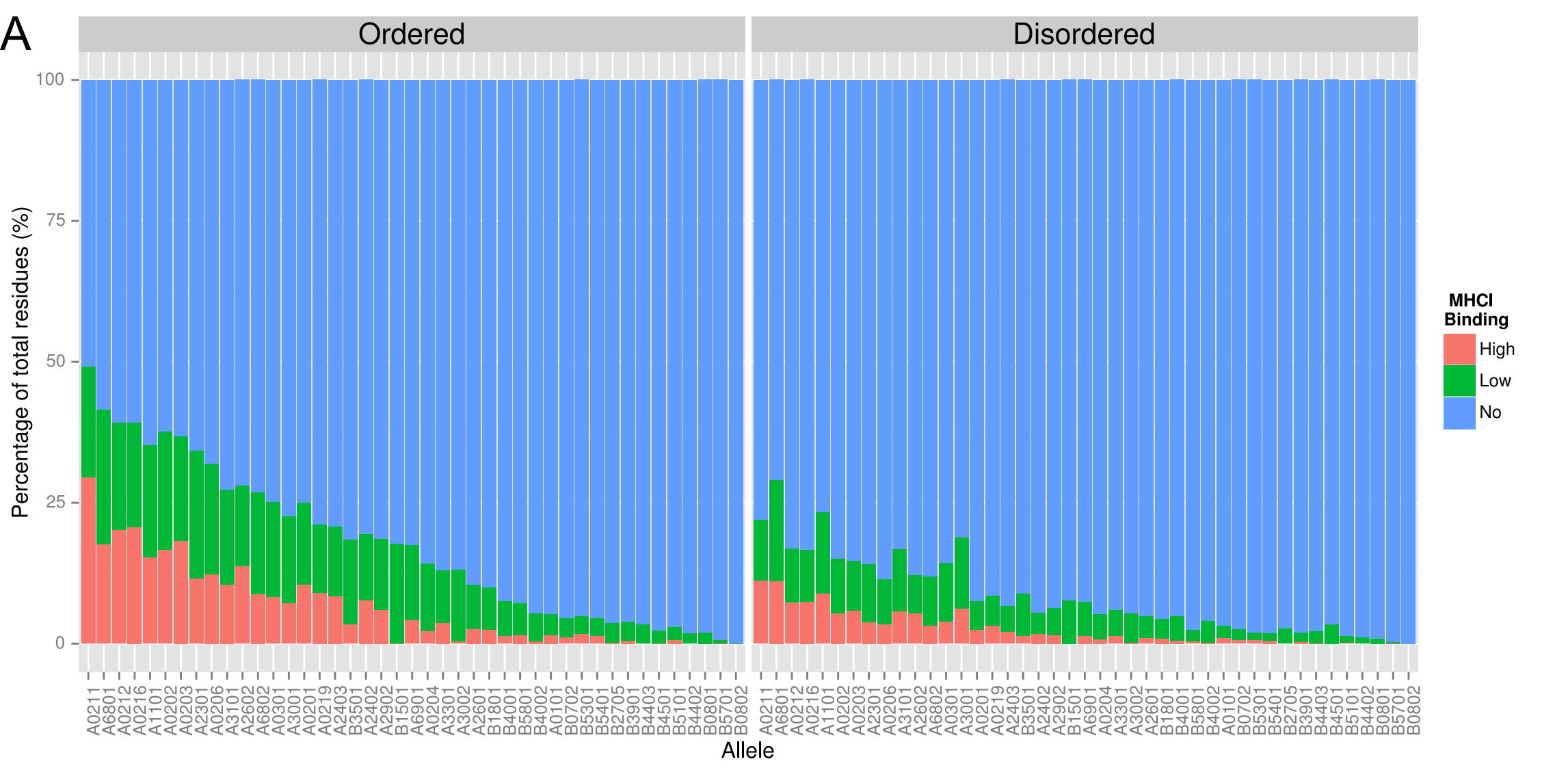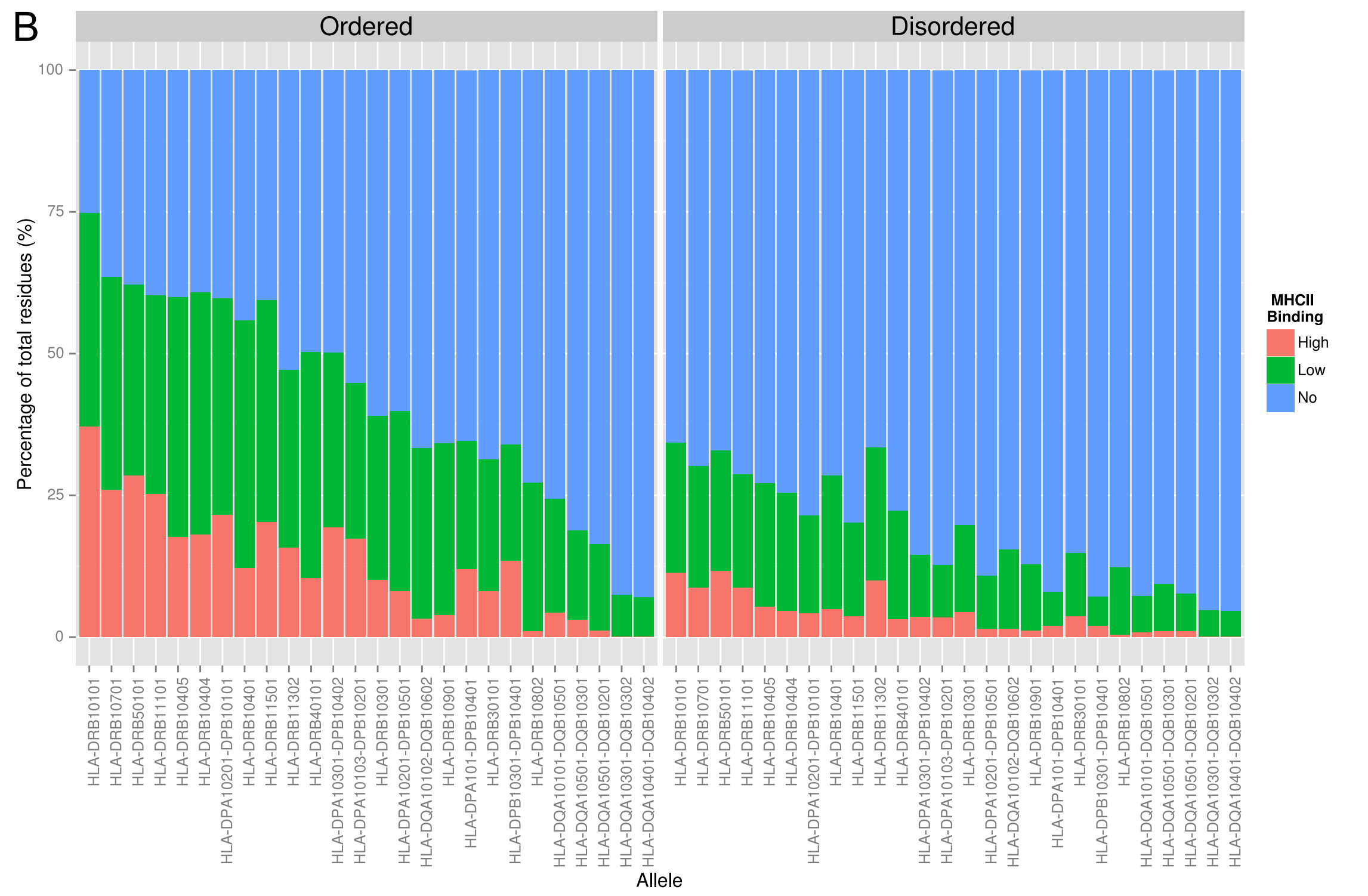

Supplement: S1 Fig — The proportion of peptides with predicted binding to MHCI (A) and MHCII (B) is significantly higher for peptides that are contained within a structured protein domain. Prediction of protein disorder was performed using DISOPRED3, while predictions of MHC class I and MHC class II binding were performed with NetMHC 3.0 and NetMHCII 2.2 Peptides were grouped according to their predicted binding affinity (IC50): High-affinity, IC50<50nM; Low-affinity, 50nM<IC50<500nM; No-binding, IC50>500nM. (PDF) [file pone.0141729.s001.pdf]

A

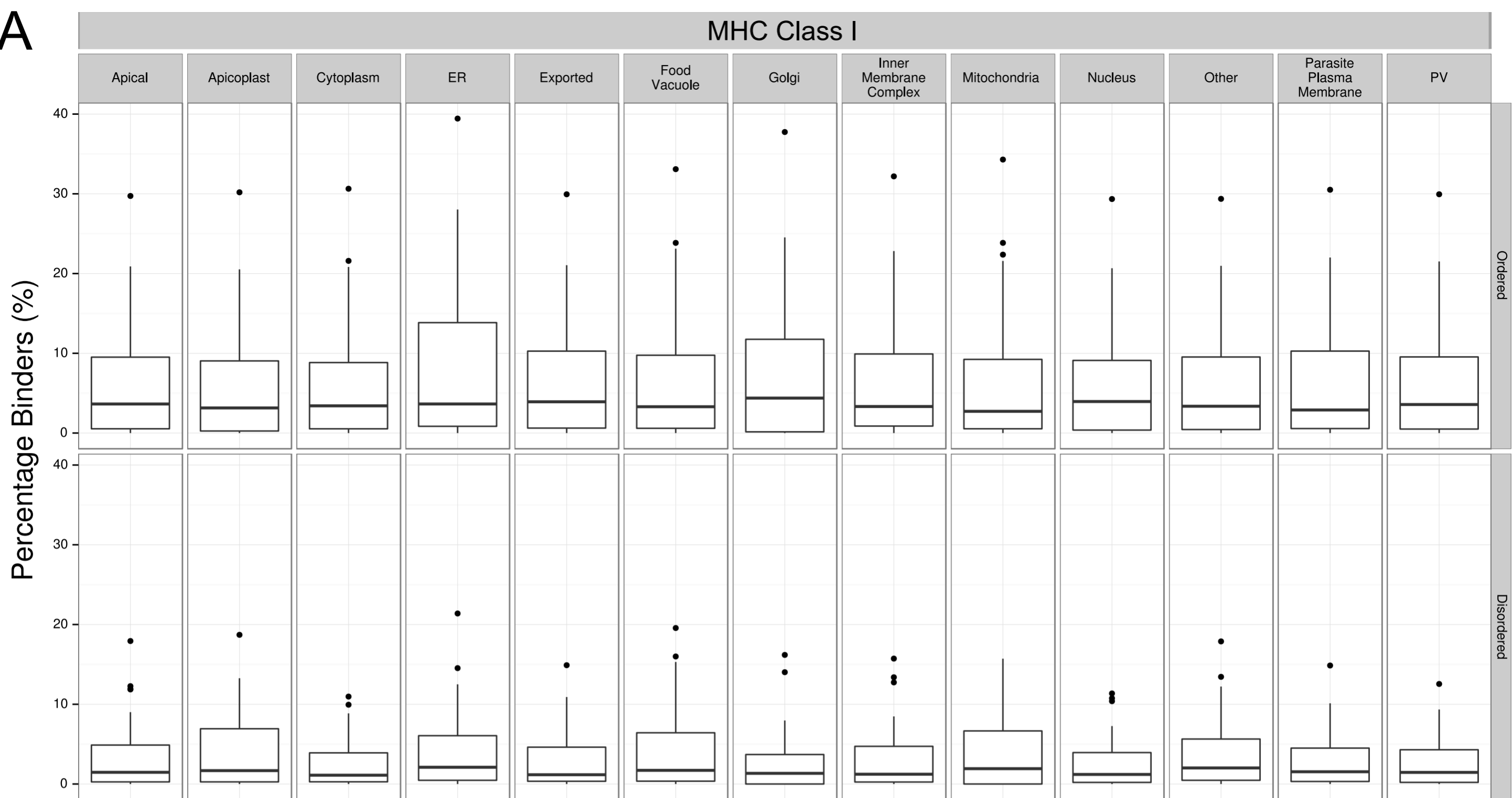

B

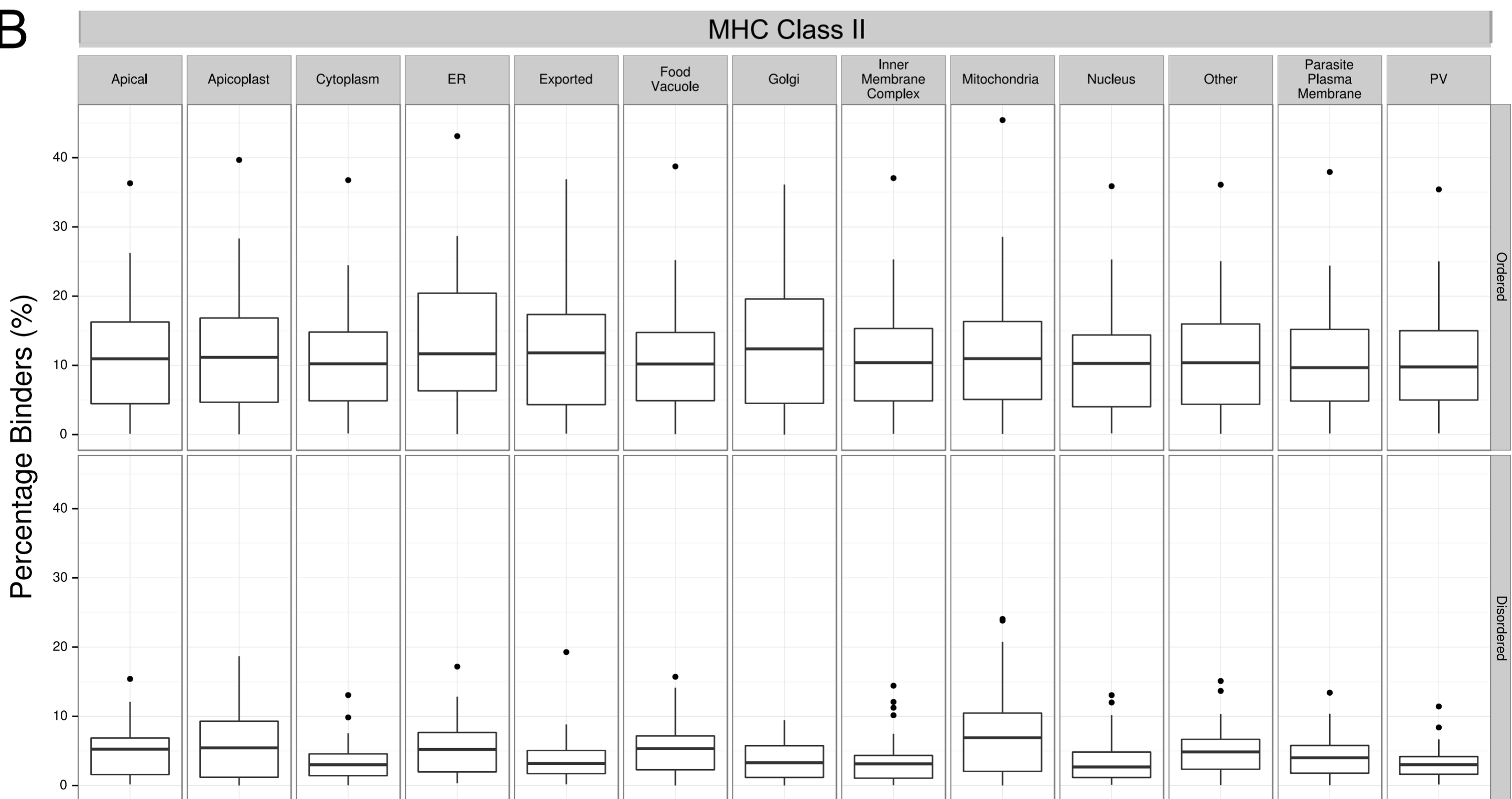

Supplement: S2 Fig — No significant difference in the proportion of MHCI (A) or MHCII (B) binding peptides was observed between different subcellular locations (p > 0.05, kruskal-wallis rank sum test). Boxplots represent the distribution of MHC-binding peptides across all MHC alleles tested. Prediction of protein disorder was performed using DISOPRED3, while prediction of MHC class I and MHC class II binding was performed with NetMHC 3.0 and NetMHCII 2.2. Peptides with predicted high binding affinity are shown (IC50<50nM). (PDF) [file pone.0141729.s002.pdf]

**A**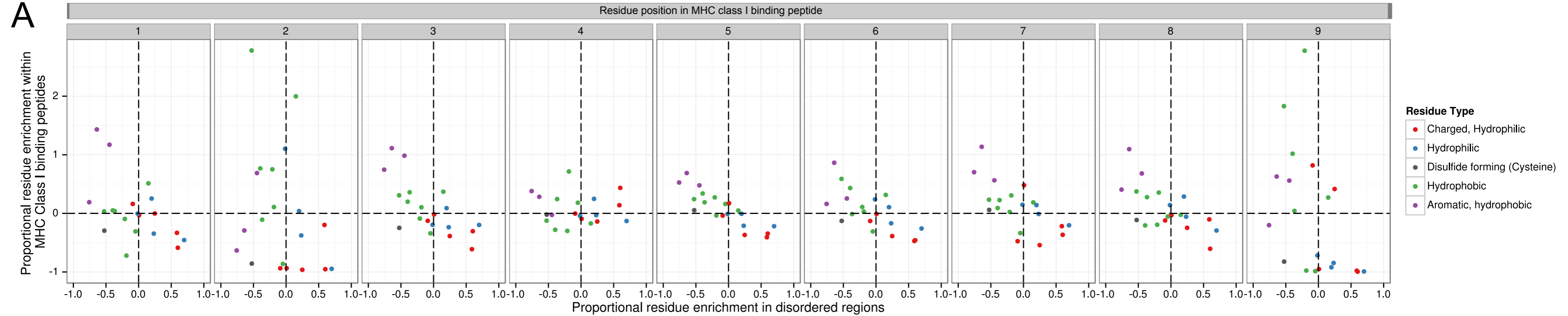**B**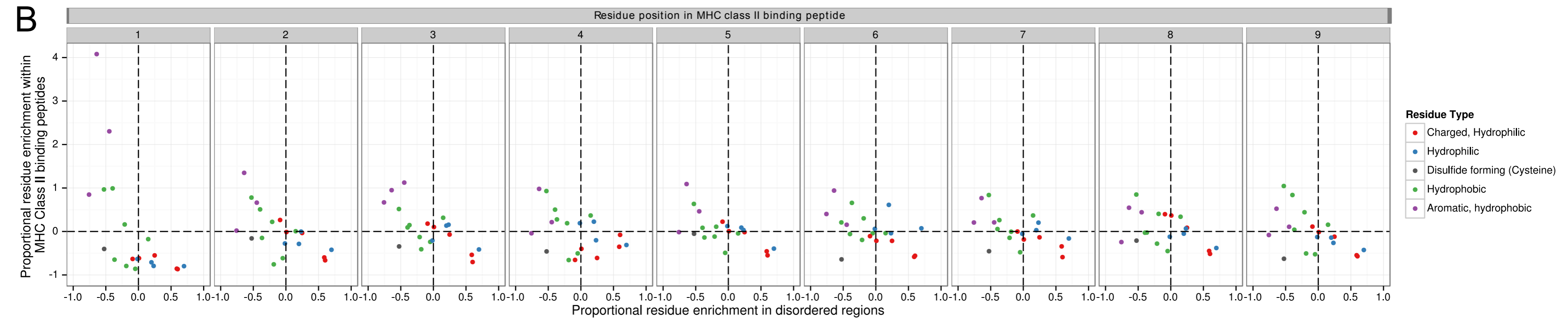

Supplement: S3 Fig — The position specific enhancement of each residue in both MHC class I (A) and MHC class II (B) binding peptides (IC50 < 50nM) was plotted against the proportional enrichment of that residue in disordered regions. Prediction of protein disorder was performed using DISOPRED3, while prediction of MHC class I and MHC class II binding was performed with NetMHC 3.0 and NetMHCII 2.2. (PDF) [file pone.0141729.s003.pdf]

**A**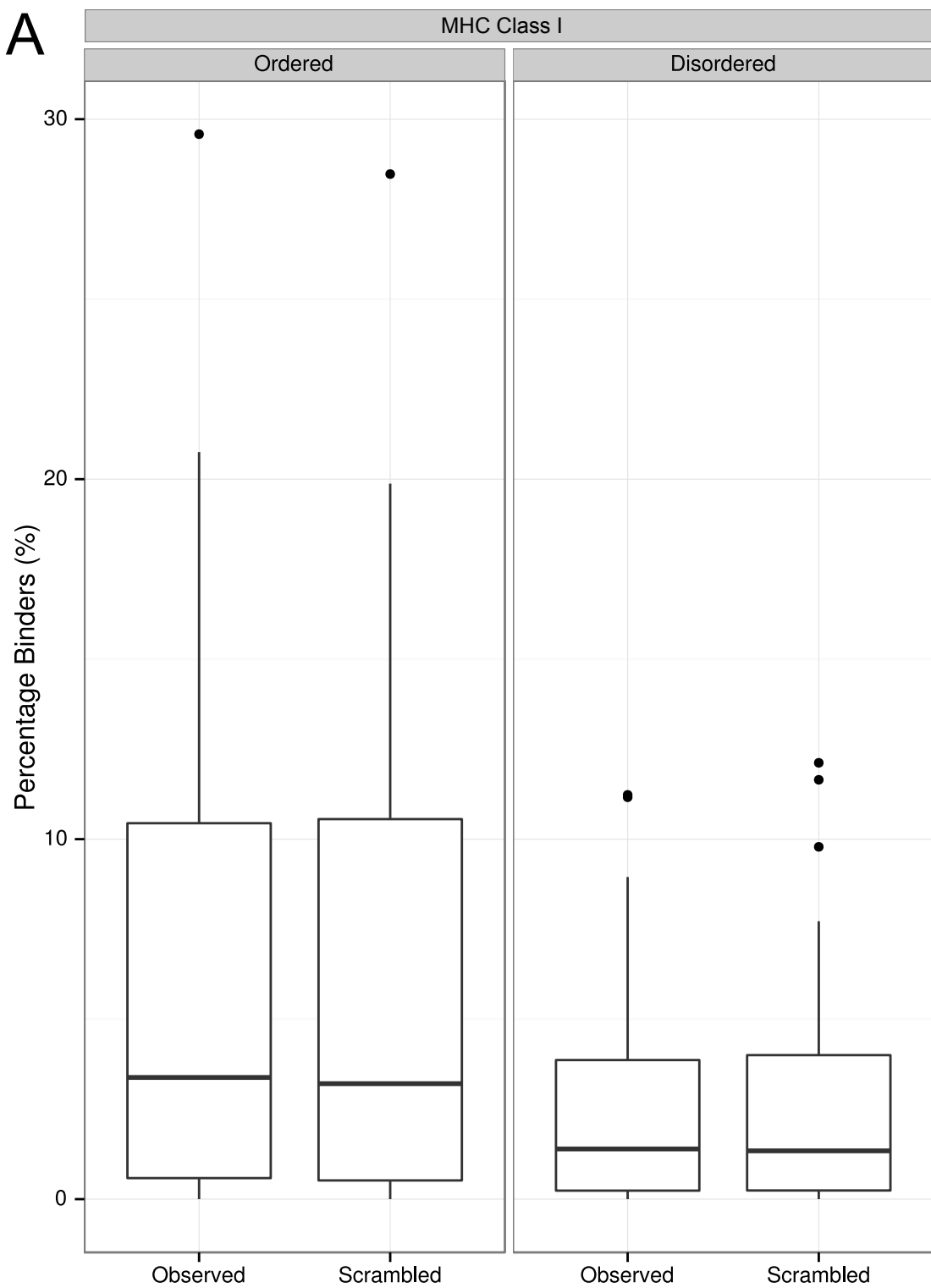**B**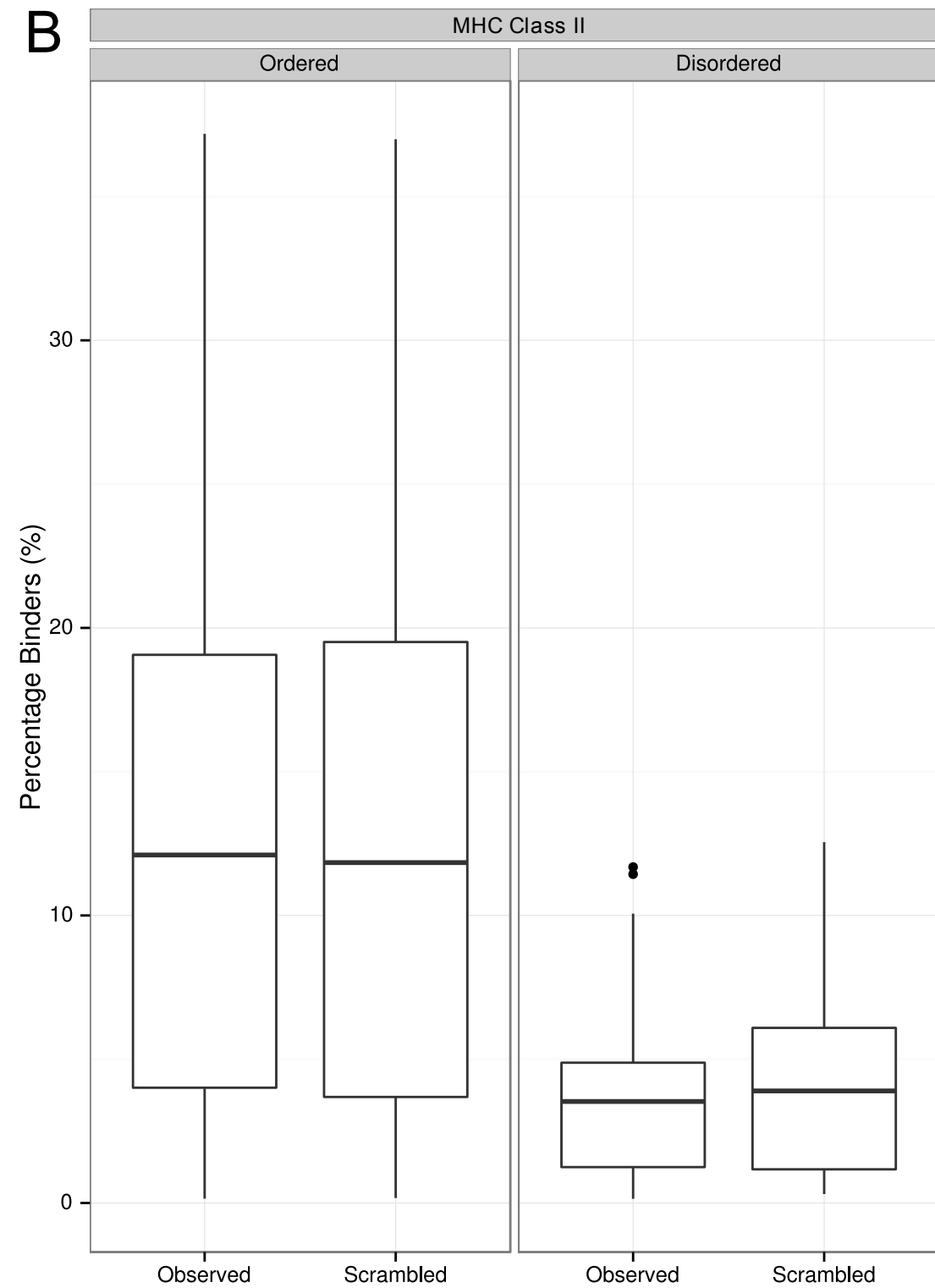

Supplement: S4 Fig — Sequences within disordered and ordered regions of each P. falciparum protein were scrambled, and the resultant scrambled proteome was submitted to predictors of MHC class I (A) and MHC class II (B) binding. Sequence scrambling was performed 4x, with results from MHC predictors averaged across all repeats. Prediction of disorder was performed with DISOPRED3. (PDF) [file pone.0141729.s004.pdf]

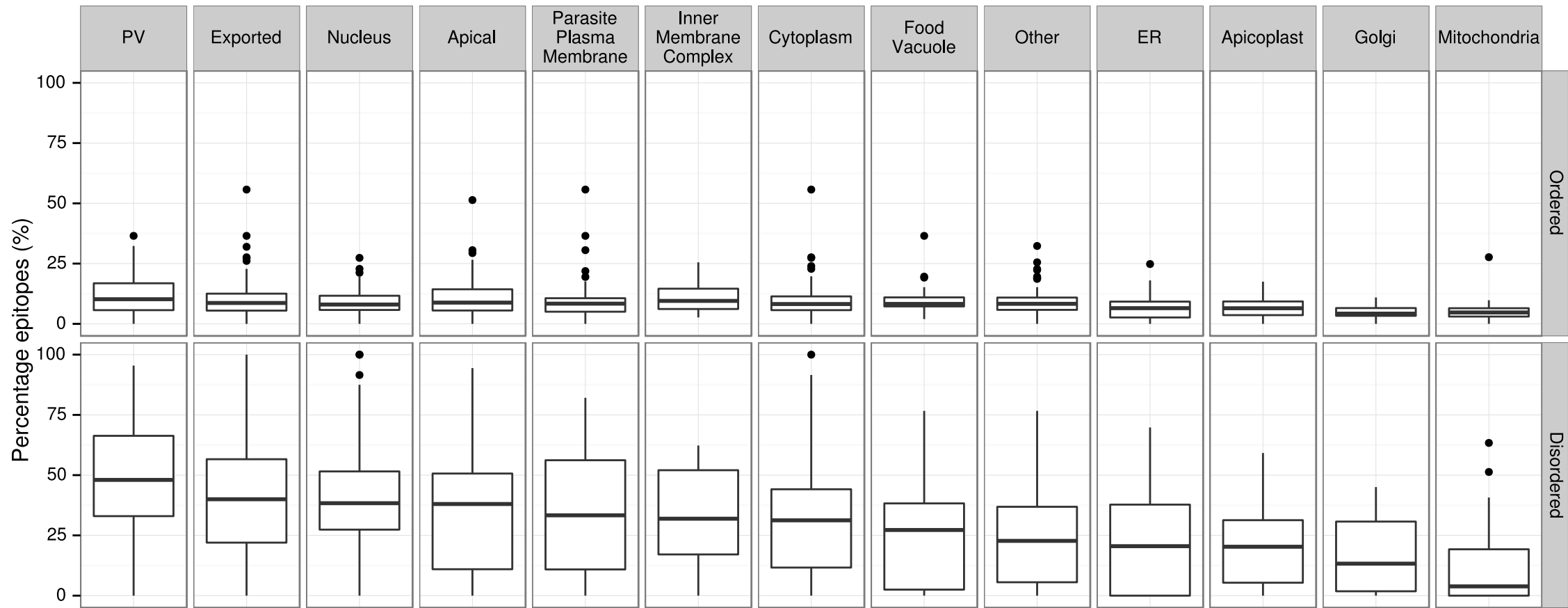

Supplement: S5 Fig — Classification of disorder was achieved using DISOPRED3. BepiPred was used for prediction of linear B-cell epitopes. A threshold of 0.9 was used for BepiPred predictions. Protein localisation was classified using the ApiLoc resource. A total of 451 proteins were assigned a location. (PDF) [file pone.0141729.s005.pdf]
