# Supplementary material for: Insights into the Immunological Properties of Intrinsically Disordered Malaria Proteins Using Proteome Scale Predictions
Source: PLoS One. 2015 Oct 29;10(10):e0141729. doi: 10.1371/journal.pone.0141729 (PMC4626106; doi:10.1371/journal.pone.0141729)
Supplement: S1 Table — Protein localisation was classified using the ApiLoc resource. Prediction of disorder was performed using DISOPRED3. A total of 451 proteins were assigned a location. Percentage disorder was calculated as the proportion of residues predicted to be disordered at the level of individual proteins. (DOCX) [file pone.0141729.s007.docx]

**Table S1:** **Summary statistics for predicted protein disorder of *P. falciparum* proteins, grouped according to subcellular localisation.** Protein localisation was classified using the ApiLoc resource. Prediction of disorder was performed using DISOPRED3. A total of 451 proteins were assigned a location. Percentage disorder was calculated as the proportion of residues predicted to be disordered at the level of individual proteins.

| Location | Median | IQR | W statistic | df | p-value |
| --- | --- | --- | --- | --- | --- |
| Apical | 24.3 | 38.9 | 273081.5 | 82 | 0.0003 |
| PV | 27.7 | 29.1 | 172672 | 47 | <0.0001 |
| Exported | 28.0 | 34.1 | 285521 | 80 | <0.0001 |
| Nucleus | 28.1 | 28.7 | 248017 | 73 | 0.0002 |
| Inner Membrane Complex | 18.1 | 25.1 | 52308 | 17 | 0.51 |
| Golgi | 14.1 | 33.3 | 25671 | 9 | 0.84 |
| Parasite Plasma Membrane | 22.2 | 37.6 | 187876.5 | 62 | 0.11 |
| Cytoplasm | 15.4 | 26.3 | 266415.5 | 102 | 0.59 |
| Other | 11.4 | 24.8 | 146830.5 | 60 | 0.19 |
| Apicoplast | 15.7 | 11.0 | 99806.5 | 39 | 0.48 |
| Food Vacuole | 12.0 | 16.8 | 69140.5 | 27 | 0.50 |
| Mitochondria | 8.8 | 9.2 | 55932.5 | 28 | 0.01 |
| ER | 8.9 | 14.0 | 67192.5 | 31 | 0.04 |
