# Supplementary material for: Insights into the Immunological Properties of Intrinsically Disordered Malaria Proteins Using Proteome Scale Predictions
Source: PLoS One. 2015 Oct 29;10(10):e0141729. doi: 10.1371/journal.pone.0141729 (PMC4626106; doi:10.1371/journal.pone.0141729)
Supplement: S2 Table — Protein localisation was classified using the ApiLoc resource. A total of 451 proteins were assigned a location. A Wilcoxon Rank-Sum test was performed on proteins from each subcellular location, comparing the percentage of residues predicted to be part of a linear B-cell epitope for each protein in that location, to the distribution within the entire P. falciparum proteome. Residues were grouped according to predicted protein disorder, and statistical analysis applied to each group (ordered/disordered). (DOCX) [file pone.0141729.s008.docx]

**Table S2: Summary statistics for percentage linear B-cell epitopes within *P. falciparum* proteins, grouped according to subcellular localisation.** Protein localisation was classified using the ApiLoc resource. A total of 451 proteins were assigned a location. A Wilcoxon Rank-Sum test was performed on proteins from each subcellular location, comparing the percentage of residues predicted to be part of a linear B-cell epitope for each protein in that location, to the distribution within the entire *P. falciparum* proteome. Residues were grouped according to predicted protein disorder, and statistical analysis applied to each group (ordered/disordered).

| Location | Disorder | Median | IQR | W statistic | df | p-value |
| --- | --- | --- | --- | --- | --- | --- |
| PV | Ordered | 10.2 | 11.2 | 163005 | 47 | 0.002 |
| PV | Disordered | 48.0 | 33.3 | 179525 | 47 | <0.0001 |
| Exported | Ordered | 8.7 | 7.0 | 260513 | 81 | 0.005 |
| Exported | Disordered | 40.0 | 34.6 | 261212.5 | 80 | 0.001 |
| Apical | Ordered | 8.8 | 8.8 | 274922.5 | 83 | 0.001 |
| Apical | Disordered | 38.0 | 39.7 | 249020 | 82 | 0.054 |
| Parasite Plasma Membrane | Ordered | 8.4 | 5.6 | 193397 | 62 | 0.058 |
| Parasite Plasma Membrane | Disordered | 33.3 | 45.3 | 183593 | 62 | 0.215 |
| Nucleus | Ordered | 8.0 | 5.9 | 235542 | 74 | 0.014 |
| Nucleus | Disordered | 38.4 | 24.1 | 243684.5 | 73 | 0.001 |
| Inner Membrane Complex | Ordered | 9.5 | 8.4 | 63922 | 17 | 0.020 |
| Inner Membrane Complex | Disordered | 31.9 | 34.9 | 50334.5 | 17 | 0.733 |
| Food Vacuole | Ordered | 8.3 | 3.7 | 92857 | 27 | 0.035 |
| Food Vacuole | Disordered | 27.2 | 35.8 | 67155.5 | 27 | 0.347 |
| Cytoplasm | Ordered | 8.2 | 5.7 | 325156.5 | 106 | 0.024 |
| Cytoplasm | Disordered | 31.3 | 32.5 | 267458.5 | 102 | 0.620 |
| Other | Ordered | 8.3 | 5.1 | 195127 | 61 | 0.023 |
| Other | Disordered | 22.7 | 31.3 | 134864 | 60 | 0.020 |
| Golgi | Ordered | 4.2 | 3.2 | 19591.5 | 10 | 0.052 |
| Golgi | Disordered | 13.3 | 28.9 | 17258 | 9 | 0.052 |
| ER | Ordered | 6.5 | 6.6 | 77775 | 31 | 0.337 |
| ER | Disordered | 20.5 | 37.8 | 64689 | 31 | 0.017 |
| Apicoplast | Ordered | 6.5 | 5.7 | 97777 | 39 | 0.309 |
| Apicoplast | Disordered | 20.3 | 25.9 | 78019.5 | 39 | 0.003 |
| Mitochondrian | Ordered | 4.7 | 3.5 | 54307.5 | 28 | 0.005 |
| Mitochondrian | Disordered | 3.8 | 19.2 | 40344 | 28 | <0.0001 |
