# Supplementary material for: Insights into the Immunological Properties of Intrinsically Disordered Malaria Proteins Using Proteome Scale Predictions
Source: PLoS One. 2015 Oct 29;10(10):e0141729. doi: 10.1371/journal.pone.0141729 (PMC4626106; doi:10.1371/journal.pone.0141729)
Supplement: S3 Table — Protein localisation was classified using the ApiLoc resource. Prediction of tandem repeats was performed using TREKS, with a PSIM cutoff of 0.8. A total of 451 proteins were assigned a location. Percentage tandem repeats was calculated as the proportion of residues predicted to be part of a tandem repeat at the level of individual proteins. A Wilcoxon Rank-Sum test was performed on proteins from each subcellular location, comparing the percentage tandem repeats for proteins within each respective location to the distribution of percentage tandem repeats within the entire P. falciparum proteome. (DOCX) [file pone.0141729.s009.docx]

**Table S3:** **Summary statistics for predicted tandem repeats within *P. falciparum* proteins, grouped according to subcellular localisation.** Protein localisation was classified using the ApiLoc resource. Prediction of tandem repeats was performed using TREKS, with a PSIM cutoff of 0.8. A total of 451 proteins were assigned a location. Percentage tandem repeats was calculated as the proportion of residues predicted to be part of a tandem repeat at the level of individual proteins. A Wilcoxon Rank-Sum test was performed on proteins from each subcellular location, comparing the percentage tandem repeats for proteins within each respective location to the distribution of percentage tandem repeats within the entire *P. falciparum* proteome.

| Location | Mean | SD | Median | IQR | W statistic | df | p-value |
| --- | --- | --- | --- | --- | --- | --- | --- |
| Exported | 9.61 | 18.58 | 0.61 | 10.51 | 257829 | 81 | 0.004 |
| PV | 8.54 | 17.69 | 1.67 | 9.89 | 152788.5 | 47 | 0.016 |
| Parasite Plasma Membrane | 5.62 | 12.48 | 0.00 | 6.63 | 177916 | 62 | 0.479 |
| Apical | 5.43 | 11.97 | 0.00 | 5.62 | 244030 | 83 | 0.178 |
| Cytoplasm | 3.79 | 10.92 | 0.00 | 2.79 | 266778.5 | 106 | 0.125 |
| Nucleus | 3.38 | 6.26 | 0.00 | 4.86 | 212537 | 74 | 0.406 |
| Other | 2.70 | 9.48 | 0.00 | 0.00 | 140416.5 | 61 | 0.014 |
| Golgi | 2.14 | 7.11 | 0.00 | 0.00 | 21433.5 | 10 | 0.072 |
| Food Vacuole | 1.98 | 4.46 | 0.00 | 0.89 | 67304 | 27 | 0.258 |
| ER | 1.84 | 4.75 | 0.00 | 0.42 | 72449 | 31 | 0.075 |
| Inner Membrane Complex | 1.41 | 2.84 | 0.00 | 1.29 | 41423.5 | 17 | 0.222 |
| Apicoplast | 0.61 | 1.70 | 0.00 | 0.00 | 81310 | 39 | 0.002 |
| Mitochondrian | 0.39 | 1.78 | 0.00 | 0.00 | 51823 | 28 | 0.0004 |
