# Supplementary material for: Insights into the Immunological Properties of Intrinsically Disordered Malaria Proteins Using Proteome Scale Predictions
Source: PLoS One. 2015 Oct 29;10(10):e0141729. doi: 10.1371/journal.pone.0141729 (PMC4626106; doi:10.1371/journal.pone.0141729)
Supplement: S4 Table — Protein localisation was classified using the ApiLoc resource. A total of 451 proteins were assigned a location. A Wilcoxon Rank-Sum test was performed on proteins from each subcellular location, comparing the percentage of residues targeted by non-synonymous SNPs for each protein in that location, to the distribution of SNPs within the entire P. falciparum proteome. (DOCX) [file pone.0141729.s010.docx]

**Table S4: Summary statistics for SNPs within *P. falciparum* proteins, grouped according to subcellular localisation.** Protein localisation was classified using the ApiLoc resource. A total of 451 proteins were assigned a location. A Wilcoxon Rank-Sum test was performed on proteins from each subcellular location, comparing the percentage of residues targeted by non-synonymous SNPs for each protein in that location, to the distribution of SNPs within the entire *P. falciparum* proteome.

| Location | Mean | SD | Median | IQR | W statistic | df | p-value |
| --- | --- | --- | --- | --- | --- | --- | --- |
| Exported | 1.67 | 3.71 | 0.46 | 1.17 | 268938.5 | 81 | 0.001 |
| PV | 1.47 | 3.14 | 0.60 | 1.08 | 164441.5 | 47 | 0.001 |
| Apical | 1.46 | 2.65 | 0.55 | 1.27 | 290436.5 | 83 | <0.0001 |
| Parasite Plasma Membrane | 1.22 | 1.91 | 0.32 | 1.72 | 193234.5 | 62 | 0.054 |
| ER | 0.83 | 2.46 | 0.19 | 0.48 | 83620 | 31 | 0.744 |
| Nucleus | 0.60 | 2.30 | 0.00 | 0.37 | 174068 | 74 | 0.030 |
| Inner Membrane Complex | 0.57 | 1.53 | 0.17 | 0.37 | 43346 | 17 | 0.410 |
| Cytoplasm | 0.54 | 1.19 | 0.00 | 0.42 | 254718 | 106 | 0.029 |
| Food Vacuole | 0.47 | 0.67 | 0.27 | 0.43 | 79508 | 27 | 0.624 |
| Golgi | 0.45 | 0.79 | 0.00 | 0.41 | 26268.5 | 10 | 0.491 |
| Other | 0.44 | 0.95 | 0.00 | 0.45 | 142575 | 61 | 0.037 |
| Mitochondrian | 0.19 | 0.46 | 0.00 | 0.16 | 52161.5 | 28 | 0.001 |
| Apicoplast | 0.11 | 0.18 | 0.00 | 0.17 | 75237.5 | 39 | 0.001 |
